# The *Kenny music performance anxiety inventory* (K-MPAI): Scale construction, cross-cultural validation, theoretical underpinnings, and diagnostic and therapeutic utility

**DOI:** 10.3389/fpsyg.2023.1143359

**Published:** 2023-05-26

**Authors:** Dianna Theadora Kenny

**Affiliations:** ^1^DK Consulting, Sydney, NSW, Australia; ^2^The University of Sydney, Sydney, NSW, Australia

**Keywords:** Kenny music performance anxiety inventory, music performance anxiety, musicians, cross-cultural validation, factor structure, performance psychology

## Abstract

I commenced my academic exploration of music performance anxiety in a study with opera chorus artists from Opera Australia in 2004. I subsequently postulated a new theory of the aetiology of music performance anxiety and began the development of the *Kenny Music Performance Anxiety Inventory* (K-MPAI) to assess the hypothesized theoretical constructs underpinning its diverse clinical presentations. I proposed a new definition of music performance anxiety in 2009 and revised the item content of the K-MPAI from 26 to 40 in 2011. Over the ensuing years, many researchers have used the K-MPAI in studies on a wide variety of musicians, including vocalists and instrumentalists, popular and classical musicians, tertiary music students, and professional, solo, orchestral, ensemble, band, and community musicians. To date, the K-MPAI has been reported in more than 400 studies and has been translated into 22 languages. It has been the subject of more than 39 dissertations. In this paper, I examine the research that has used the K-MPAI to assess the theory and to ascertain how well the assessment tool, and its cross-cultural validation have provided evidence for its factorial structure, robustness, and utility. The evidence indicates that the factorial structure remains consistent across cultures and different populations of musicians. It has good discriminative ability and utility for diagnostic purposes. I conclude with some reflections on how the K-MPAI can guide therapeutic interventions and with some thoughts on future directions.

## 1. Construction of the *Kenny music performance anxiety inventory* (K-MPAI)

*Nothing is more devastating to a performing artist than not having the chance to be on stage and, as the pervasiveness of performance anxiety attests, nothing is more threatening than having that chance* (Plaut, 1990).

The *Kenny Music Performance Anxiety Inventory* (K-MPAI; Kenny, 2009) is a 40-item inventory that assesses an emotion-based theory of anxiety (Barlow, 2000, 2004) as it applies to anxiety in the context of music performance. Items address each of Barlow’s theoretical constructs that underpin anxiety–evocation of anxious propositions (e.g., uncontrollability, unpredictability, negative affect, situational cues); attentional shift (e.g., task or self-evaluative focus, fear of negative evaluation); physiological arousal, and memory. Items are assessed using a seven-point Likert scale, with higher scores indicating more severe MPA and psychological distress generally (including depression).

The first studies of the factorial structure of the K-MPAI were undertaken with 379 professional orchestral musicians in Australia and 159 tertiary music students in New Zealand. For the orchestral musicians, exploratory factor analysis with varimax rotation produced six factors–proximal somatic anxiety and worry about performance (*α*= 0.91); worry/dread (negative cognitions/ruminations) focused on self/other scrutiny (*α*= 0.86); depression/hopelessness (psychological vulnerability)(*α* = 0.85); parental empathy (*α*= 0.75); concerns with memory (*α* = 0.92); generational transmission of anxiety (*α* = 0.72); an additional weaker factor – anxious apprehension (*α* = 0.59); and one item for biological vulnerability (Kenny et al., 2012). For the tertiary level music students, Cronbach’s alpha for internal consistency (*α*) was 0.94 (Kenny, 2009). There were 12 underlying factors, which could be subsumed under the following three meta-factors: 1. Early relationship context: (7) generational transmission of anxiety; (4) parental empathy]; 2. Psychological vulnerability: [(1) depression/hopelessness (9); controllability; (11) trust (12); pervasive performance anxiety]; and 3. Proximal performance concerns: [(3) proximal somatic anxiety; (2) worry/dread (negative cognitions); (6) pre- and post-performance rumination; (8) self/other scrutiny; (10) opportunity cost; (5) memory reliability] (Kenny, 2011).

Receiver operating curves were generated using established clinical screening tests [State–Trait Anxiety Inventory – Trait (STAI-T) (Spielberger, 1983), PRIME-MD (Spitzer et al., 2003), Social Phobia Inventory (SPIN) (Connor et al., 2000)] validated in clinical populations to identify clinical cut-off scores for the K-MPAI. The cut-point for K-MPAI using Youden’s Index for STAI-T ≥ 65 (1.5 SD above mean) was 105.3; for STAI-T ≥ 60 (1 SD above mean), Youden’s Index for K-MPAI was 104.5. For musicians answering yes to both depression questions on the PRIME-MD, the K-MPAI cut-point was 118.5; if they answered “yes” to one of two questions, K-MPAI cut-point was 110. As previously identified, K-MPAI and SPIN were unrelated (Kenny, 2016a).

The K-MPAI has been used in studies of tertiary level music students (e.g., Kenny et al., 2013; Paliaukiene et al., 2018; Rauf and Laitf, 2018; Oh et al., 2020); amateur musicians (Barbar et al., 2015); community musicians (Kenny and Halls, 2018); school band directors (Yoder, 2022); ensemble musicians (Robson and Kenny, 2017); elite orchestral musicians (Kenny et al., 2012, 2016, 2018; Kenny and Ackermann, 2015), opera chorus artists (Kenny et al., 2004); popular musicians (Bober, 2019); and Indian rock musicians (Meitei and Kumari, 2014).

The K-MPAI has also been modified for use with performers in fields other than music (Kantor-Martynuska and Kenny, 2018). In this study, the Polish translation of the K-MPAI was modified and named the *Kenny Performance Anxiety Inventory* (K-PAI). On a sample of 586 performing artists, the performance of the K-PAI was assessed using measures of general anxiety, depression, attentional control, and reward susceptibility. The scores on K-PAI revealed strong associations with trait anxiety and depression and negative associations with attentional control and susceptibility to reward. These results replicated those obtained on the K-MPAI with Australian musicians, indicating the cross-cultural validity of the K-MPAI and K-PAI. We concluded that performance anxiety develops on the basis of biological and psychological predispositions and early negative experiences in performance contexts.

The K-MPAI has been translated into 22 languages–Brazilian Portuguese (Rocha et al., 2011), Croatian (Ružak, 2021; Eva Stevanovic, 2022, personal communication), Czech (Eva Stevanovic, 2022, personal communication; Pavel Husa Fel, 2022, personal communication), Dutch (van Fenema et al., 2017), French (Antonini Philippe et al., 2022b), German (Peschke and von Georg, 2015), Hungarian (Dobos et al., 2019), Indonesian (Haninditya, 2021), Italian (Antonini Philippe et al., 2022a), Japanese (Sakie Takagi, Michiko Yoshie, Akihiko Murai, 2023 Korean (Oh et al., 2020), Latvian (Solveiga Sofija Saulīte, 2022, personal communication), Lithuanian (Paliaukiene and Kairys, 2012), Mandarin (Simplified Chinese) (Diana Wu, personal communication, Cancan Cui, personal communication); Persian (Fakhr, 2020; Kbodadadeh et al., 2022), Polish (Kantor-Martynuska and Kenny, 2018), Portuguese (Rocha et al., 2011), Slovenian Kaja Pojbič, Ana Gregorec, Urban Stiberc and Zala Brecko (personal communication, 2022), Spanish (also for Peru) (Chang-Arana, 2015; Zarza-Alzugaray et al., 2015; Chang-Arana, 2017; Chang-Arana et al., 2018), Taiwanese (Lin, 2019), Romanian (Faur et al., 2021), Turkish (Rocha et al., 2011; Çiçek and Güdek, 2020), and Ukrainian (Ksondzyk, 2020). I am advised that a Finnish translation is currently in preparation. All currently translations of the K-MPAI are available in Supplementary material. To date, the K-MPAI has been the subject of around 400 studies (see Supplementary material), and 39 doctoral dissertations were located (Note: This may not be an exhaustive list) (see Supplementary material for available translations).

## 2. Cross-cultural validation of the K-MPAI

In this review, I have excluded those studies that did not use the K-MPAI in a standardized way, for example, by pre-selecting items to shorten the questionnaire without explanation for item choice, or that have altered the K-MPAI in some way, for example, by changing the seven-point Likert scale to a dichotomous category (Always/Never) (Kbodadadeh et al., 2022). Sadly, I also could not include studies in most languages other than English that did not provide an abstract or summary in English or French. These are studies that could benefit from English-language translation to make their findings more widely available.

Some studies have used the 26-item version of the K-MPAI (e.g., Barbar et al., 2015; Zarza-Alzugaray et al., 2015; Casanova et al., 2018; Ksondzyk, 2020; Mancin et al., 2022), and although not directly comparable to the 40-item version, make important contributions. For example, Barbar et al. (2015), using a Portuguese Brazilian translation of the K-MPAI of the 26-item version with 230 graduate or undergraduate level amateur musicians (58% female; mean age = 39.17 years – SD = 16.48) and exploratory factor analysis, identified three factors that together accounted for 62% of the total shared variance. These were 1. Worries and insecurity (*α* = 0.82), 2. Depression and hopelessness (*α* = 0.77), and 3. Early parental relationships (*α* = 0.57). These themes repeatedly recur in subsequent studies using the 40-item version. In the 26-item version, items were specifically chosen to assess each of the three dimensions of Barlow’s emotion-based theory (Kenny, 2009).

(i) Biological vulnerability predispositions (e.g., Behavioral inhibition, autonomic reactivity) and early contextual/parental vulnerabilities: items 5, 9, 19, 21, 24
(ii) Generalized psychological vulnerability: items 1, 2, 3, 4, 6, 8, 10, 11, 15, 16, 17, 18, 23
(iii) Specific triggering factors causing subsequent concerns about performance: items 7, 12, 13, 14, 20, 22, 25, 26.

The 40-item version retains this structure while enhancing some of these dimensions with the Addition 14 items.

After studies confirmed the validity of the English and Spanish versions of the K-MPAI (Chang-Arana, 2015; Chang-Arana, 2017), further exploration of the factorial structure was undertaken, applying higher order exploratory factor analysis (HOEFA) and an invariance analysis (Chang-Arana et al., 2018). Participants were 455 Peruvian tertiary music students, 74% male, mean age 21.19 years (SD = 3.13; range = 18–40 years) and the Australian sample of orchestral musicians described above, of whom 40% were male, mean age 42.07 years (SD = 10.21, range = 18–68 years). The model identified two first order factors–1. Music performance anxiety (*α* = 0.91) and 2. Depression (*α* = 0.81)–and one higher order factor that we named “negative affectivity in relation to music performance (*α* = 0.92).” These three factors explained 59% of common shared variance. The invariance analysis demonstrated similar structure and interpretation of K-MPAI scores in both populations despite their cultural, age, and musical status differences. The factorial structure obtained supported a unidimensional interpretation of the construct of MPA related to negative affectivity (depression), not anxiety. This study provides compelling support for Kenny’s theoretical conceptualization of MPA as a complex psychological disorder particularly for those musicians experiencing more severe forms of MPA.

Recently, several studies have been published that further explore the factorial structure of the K-MPAI in various populations and languages. Table 1 provides a summary of the outcomes of this research. Most have provided confirmation of the factorial structure of the K-MPAI found in English-speaking musicians and strong support for the underlying theoretical structure on which the inventory was constructed.

**Table 1:** Cross-cultural studies of the factor analysis of the KMPAI.

Authors	Language	Sample	Reliability	Factors	Variance explained	Items excluded (criterion for exclusion)
Antonini Philippe et al. (2022a)	Italian	419 music conservatoire students, 47% female, mean age = 23.18 years, SD = 5.26; range = 18–38 years	EFA *α* = 0.93	1. Music performance anxiety symptoms (*α* = 0.93)	46%	2,7,8,24,27,39 (factor loading<0.4)
				2. Depression and hopelessness (*α* = 0.86)		
				3. Parental support (*α* = 0.69)		
				4. Memory self-efficacy (*α* = 0.89)		
				5. Generational transmission of anxiety (*α* = 0.79)		
Antonini Philippe et al. (2022b)	French	211 music students from music schools and colleges in French-speaking Switzerland, 55% female, mean age = 25.34 years, SD = 9.58 (range16-65 years).	CFA *α* = 0.91	1. Proximal somatic and cognitive anxiety (*α* = 0.87)	39%	2, 5, 8, 11, 17, 28, 29, 32,40 (factor loading<0.3)
				2. Self/other scrutiny and evaluation (*α* = 0.78)		
				3. Psychological vulnerability (*α* = 0.80)		
				4. Confidence in memory (*α* = 0.78)		
				5. Early parental relationship context (*α* = 0.61)		
Dias et al. (2022)	Portuguese Portugal	164 undergraduate music students in Portugal (62.2% female; mean age = 22.63; SD = 4.36)	EFA *α* = 0.91	1. Music performance anxiety-related symptoms	51%	1,2,5,7,8,17,29,31,32,39,40
				2. Depression and hopelessness		
				3. Parental support		
				4. Memory self-efficacy		
Mancin et al. (2022)	Italian 26 items	319 music performers 28% female, mean age = 25.51; SD = 8.1(range 8 and 62 years)	EFA CFA (*α* = 0.87)	1. Proximal performance concerns (*ω* = 0.86)	40%	2,7,8
				2. Helplessness – psychological vulnerability (*ω* = 0.86)		
				3. Early parental context and memory reliance (*ω* = 0.80)		
Faur et al. (2021)	Romanian	420 musicians, 48% female, mean age = 24.46, SD = 7.36 (range = 18–66 years)	EFA (*α* = 0.91)	1. Music performance anxiety-related symptoms (*α* = 0.93)	49%	1,2,5,7,8,17,26,29,32,36,39,40 (factor loading<0.4)
				2. Parental support (*α* = 0.77)		
				3. Depression and hopelessness (*α* = 0.86)		
				4. Memory self-efficacy (*α* = 0.81)		
Ksondzyk (2020)	Ukrainian 26 items	252 Ukrainian tertiary music students and professional musicians from Lviv, Ivano, Frankivsk, Kyiv, Ternopil, Kharkiv, who worked in state music education institutions, music academies and philharmonic societies 59% female, mean age = 35.32, SD = 13.3, range 18–72 years	EFA (*α* = 0.87) CFA	1. Proximal performance concerns (*α* = 0.90)	62.8% for 7 factor model, 45% for 3 factor model	2,3,8,26 (factor loading<0.416)
				2. Psychological vulnerability (*α* = 0.83)		
				3. Early relationship context (*α* = 0.66).		
				1. Proximal somatic anxiety (*α* = 0.86)		
				2. Worry/dread (negative cognitions) (*α* = 0.82)		
				3. Depression/hopelessness (*α* = 0.74),		
				4. Anxious apprehension (*α* = 0.84)		
				5. Parental empathy (*α* = 0.65),		
				6. Generational transmission of anxiety (*α* = 0.66)		
Oh et al. (2020)	Korean	69 art high school students	EFA	1. Worry/dread and negative cognitions	61%	
				2. Proximal somatic anxiety and worry about performance		
				3. Depression/hopelessness		
				4. Parental empathy–memory controllability		
				5. Generational transmission of anxiety		
				6. Trust		
				7. Rumination		
Chang-Arana et al. (2018)	Spanish Peru	455 Peruvian tertiary music students (25% female; mean age = 21.19 years, SD = 3.13, range = 18–40 years) and 368 Australian professional orchestral musicians (51% female; mean age = 42.07 years, SD = 10.21, range = 18–68 years)	First order EFA High order EFA (HOEFA) with Min Rank Factor Analysis (MRFA)	1. Proximal performance concerns 20 items, (*α* = 0.91),	59% 59%	8,9,22,23,25, 33, 35, 37,40 (factor loading<0.3)
				2. Psychological vulnerabilities seven items, (*α* = 0.80)		
				3. Confidence in memory two items, (*α* = 0.82)		
				4. Early parental relationship context three items, (*α* = 0.71).		
				1. Negative affectivity in relation to music performance (*α* = 0.92)		
				2. Music performance anxiety (*α* = 0.91)		
				3. Depression (*α* = 0.81)		
Barbar et al. (2015)	Portuguese Brazilian	230 graduate or undergraduate level amateur musicians (58% female; mean age 39.17 years – SD = 16.48).	EFA	1. Worries and insecurity (*α* = 0.82)	62%	
				2. Depression and hopelessness (*α* = 0.77)		
				3. Early parental relationships (*α* = 0.57).		
Zarza-Alzugaray et al. (2015)	Spanish (26 items)	Tertiary music students in conservatories in Spain *n* = 215 (EFA) *n* = 275 (CFA)	EFA CFA	Seven factors – full scale α = 0.8 Three factors corresponding to Barlow (2000) and Kenny (2009).	58%	2,8,19 (factor loading<0.3) 2,5,8,26 (factor loading<0.3)
				1. Early life context (*α* = 0.57)		
				2. Psychological vulnerability (*α* = 0.79)		
				3. Specific cognitions re performance context (*α* = 0.87)		

Content, construct, convergent, and discriminative validity, and clinical utility of the Spanish, Portuguese, and Portuguese Brazilian adaptations of the K-MPAI have been demonstrated in studies of Brazilian (Rocha et al., 2011; Barbar et al., 2014a, 2015), Spanish (Zarza-Alzugaray et al., 2015; Casanova et al., 2018), Portuguese (Dias et al., 2022), and Peruvian (Chang-Arana, 2015) musicians. Rocha et al. (2011) using the Brazilian Portuguese translation of K-MPAI with 218 professional and amateur musicians from Brazil, reported very high internal consistency for the K-MPAI (*α* = 0.98), and a correlation of 0.64 with STAI-T. Most studies showed good convergent validity with other depression and anxiety scales used in general populations. Similarly, Chang-Arana et al. (2018) reported correlations between K-MPAI and STAI-Trait scores, *r* = 0.70, results that are comparable with English-speaking musicians. Two studies reported temporal stability, finding good test–test reliability for the K-MPAI (*r* = 0.87, *p* < 0.001; Ksondzyk, 2020; Mancin et al., 2022). One study also demonstrated sex invariance in the factorial structure of the K-MPAI (Mancin et al., 2022).

The K-MPAI also discriminated between music students with and without a prior history of anxiety disorders in addition to MPA (Figueiredo, 2020; Dias et al., 2022). Similarly, Wiedemann et al. (2022) showed that tertiary music students with a pathological anxiety profile consistently showed clinically relevant levels of MPA as assessed by the K-MPAI. Using a sample of 258 students from the Lithuanian Academy of Music and Theater, (65% female; mean age = 21.6, SD = 3.3, range 18 to 54 years), Paliaukiene et al. (2018) reported that students with the highest K-MPAI scores had the poorest academic achievement and the fewest number of concerts performed in the year of the study. Casanova et al. (2018), using a sample of 476 students studying for *Grado Superior de Música* in five Spanish music schools (47% female, mean age = 22.59, SD = 4.73, range 16 to 50 years) reported increasingly higher scores on K-MPAI over 4 years of study for soloists, with first year students showing less MPA than final year students, but found no such differences for students training for orchestral positions. K-MPAI has also been used to assess treatment outcomes, showing good sensitivity to effective treatment interventions (e.g., Juncos et al., 2017; Juncos and De Paivae Pona, 2018; Jelen, 2021).

## 3. Theoretical and clinical conceptualizations of MPA

Most of the theoretical concepts that I have applied to my understanding of MPA, its assessment and treatment have been derived from my clinical work with anxious musicians, my cognate disciplines of developmental psychology and developmental psychopathology, and my understanding and application of both cognitive behavioral therapies and psychodynamic psychotherapies, particularly attachment-informed and intensive short term dynamic psychotherapy. A psychodynamic understanding of MPA posits that the performance situation stirs conflicting unconscious desires, wishes, and tensions. The audience has a pivotal role in this process because of “the universal propensity of performers to experience an audience as though it were a person from childhood, real or imagined” (Weisblatt, 1986). As is the case for all causes of anxiety, music performance anxiety in its severe form is multi-determined.

Music psychology related to MPA was still in its infancy in the last century. Prior to 1994, performance anxiety was not included in the classificatory systems of psychological or psychiatric disorders. In the DSM-IV (APA, 1994) and DSM-IV-TR (APA, 2000) performance anxiety is briefly discussed in a section on differential diagnosis in social phobia. It was therefore necessary to stand on the shoulders of the giants of anxiety research in the psychological literature (e.g., Barlow, 2004) in order to formulate my theory and typology of MPA quality and severity. This formulation challenged the prevailing view that music performance anxiety (MPA) was a subtype of social anxiety (social phobia) (discussed below). It also challenged the view that MPA was a unidimensional construct occurring on a continuum of severity from career stress at the low end to stage fright at the high end (Brodsky, 1996). I argued that MPA is better understood as a typology comprising three subtypes to account for qualitative differences in clinical presentation, severity, and co-morbidities. The three subtypes are: (i) MPA as a focal anxiety, where there is no generalized or social anxiety, depression or panic and the anxiety is specifically focused on an objectively highly stressful performance such as an audition or solo recital; (ii) MPA comorbid with other anxiety disorders, in particular social anxiety disorder; and (iii) MPA with panic and depression (see Kenny, 2011, for a detailed discussion). The items in the K-MPAI are specifically constructed to assess these dimensions.

I have introduced a number of new concepts or alternate ways of understanding existing concepts (e.g., anxiety) for consideration in our conceptualization of MPA. These include taking a life course, intra-and interpersonal developmental perspective rather than a symptomatic approach to MPA presentations (although these are important for both diagnostic and therapeutic purposes), to identify attachment quality and defensive mechanisms used to shore up self-concept and self-esteem in case formulations, and to consider comorbidities in presentation, including, in particular, other anxiety disorders, depression, and somatization, and fragile personality structure (poor self-concept, low self-esteem, low self-efficacy, poor emotion regulation), as well as reconsidering the role and meaning of anxiety in music performance anxiety specifically. The challenge has been to evaluate these complex constructs heuristically *via* a questionnaire while understanding that there is no substitute for a detailed clinical interview, assessment, and formulation. I will now briefly review some key concepts relevant to MPA.

## 4. Ways of understanding anxiety

Anxiety can be understood from several perspectives, including psycho-neurobiological, physiological, and clinical, including psychodynamic and attachment-based psychotherapy. A brief description of each follows.

### 4.1. Psycho-neurobiological perspective

Anxiety is understood as an adaptive alarm during which a freeze response may occur in some threatening situations. Specifically, freezing—or tonic immobility—may overwhelm other competing action tendencies in situations where fleeing or aggressive (fight) responses are likely to be ineffective. Evolution has endowed all humans with a continuum of innate, hard-wired, automatically activated defense behaviors, called the defense cascade. Threat activates the defense cascade; flight or fight are active defense responses for dealing with threat; freezing over-rides the flight-or-fight response; tonic immobility and collapsed immobility (i.e., fainting) are responses of last resort to inescapable threat, when fear becomes overwhelming and active defense responses have failed. During this phase in the cascade, muscle tone is lost, and consciousness is compromised secondary to bradycardia-induced cerebral hypoxia. Quiescent immobility may occur after the threat has passed; it promotes rest and healing. Each of these defense reactions has a distinctive neural signature that is mediated by a common neural pathway: activation and inhibition of functional components in the amygdala, hypothalamus, periaqueductal gray, and sympathetic and vagal nuclei. The responses that make up the defense cascade are primitive emotional states—coordinated patterns of a motor-autonomic-sensory response—that may be automatically activated in the context of danger. The activation of defense responses along this cascade are generally thought to be beyond conscious control and affect muscle (i.e., somatomotor activation), viscera (visceromotor activation) and pain perception and processing in which the triggering of non-opioid analgesia blocks ascending pain signals. Freezing in humans is usually a transient state that occurs at the beginning of the threat experience. It involves heightened attention, enhanced vigilance to threat cues, and an activated, tense body poised for action. It is usually accompanied by a drop in motor activity and a decrease in heart rate. Panic and the flight impulse are closely associated with the freeze response. Porges’s polyvagal theory (Porges, 2001, 2007, 2011) added a third dimension to this conceptualization–the communication and social engagement system that has relevance to our understanding of all anxiety disorders.

### 4.2. Physiological perspective

It is important to distinguish physiological arousal (alertness, activation) which refers to the intensity of behavior that varies on a continuum from deep sleep to intense excitement or fear from somatic anxiety. Arousal is initially non-directional and may be experienced as excitement or fear. Changes in arousal levels are reflected in changes in autonomic reactivity and are experienced as elevated heart rate, blood pressure, respiration, sweating, muscle tension, indigestion, urinary frequency, increased or decreased body temperature. I have covered the subject of somatic anxiety and its management in Kenny (2011) and the interested reader is referred to that source for a more detailed discussion. I have also dealt with cognitive anxiety in the same reference, which occurs in the presence of psychological stressors, and which involves the hypothalamic–pituitary–adrenal axis. Musicians may report somatic or cognitive anxiety (anxious apprehension, fear, dread, worry, rumination, catastrophizing) alone or in concert, with each form varying along its own severity dimension. Most early studies of MPA focused on somatic anxiety experienced by musicians in situations of evaluative threat. We now know that cognitive anxiety is an additional component to MPA that is at least partially independent of somatic anxiety, i.e., one can experience high somatic anxiety and low cognitive anxiety, or low somatic anxiety and high cognitive anxiety.

### 4.3. Clinical perspectives

#### 4.3.1. Psychodynamic perspective

Freud (1926) conceptualized anxiety as both an affective signal for danger (similar to the psycho-neurobiological model) and the motivation for psychologically defending against that (perceived) danger. When an individual senses a danger situation, she is motivated to defend against the anxiety. Freud distinguished between traumatic or primary anxiety, i.e., a state of psychological helplessness in the face of overwhelmingly painful affect, such as fear of abandonment or attack, and signal or secondary anxiety, which is a form of anticipatory anxiety that alerts us to the danger of re-experiencing the original traumatic state by repeating it in a weakened form such that measures to protect against re-traumatization are enacted. In the case of musicians with performance anxiety, the danger signal relates both to early danger experiences, such as pressure and/or failure to perform well under conditions of evaluative threat, which are internalized, and current experiences of performance and performance anxiety, which are interpersonal and occur between the performer and the audience, but which are understood and interpreted within the framework of the earlier, internalized anxiety experiences. By simultaneously attending to both sets of danger experiences – the internalized past and the interpersonal present, sense can be made of the performer’s current experiences of endangerment in the performance setting.

#### 4.3.2. Attachment informed formulation

In an attachment-based formulation, anxiety acts as a defense against emotional pain erupting from re-triggered early attachment trauma in the present. The sequence is as follows: The rupture in the attachment relationship causes emotional pain in the child and a retaliatory rage toward the parent for causing the child pain. However, because the child also loves her parent, she feels guilt about experiencing rage toward someone she loves. The rage, guilt about feeling rage, grief and craving for attachment and positive feelings are all repressed into symptoms and submerged under behaviors that enable the child to continue a relationship with her parent. When the child is required to meet the needs of its primary caregivers at the expense of her own psychological development, spontaneous experience and metacognitive processes of self-reflection and the emerging sense of self is usurped and marginalized. This process eventually becomes a characteristic defensive system described as pathological accommodation (Brandchaft, 2007) or fragile character structure (Davanloo, 1995). Whenever the child is in a situation that has the potential to rupture the attachment bond, the repressed rage, guilt about the rage, grief and pain from the initial attachment rupture is re-triggered. Anxiety is experienced to block the feelings from entering conscious awareness. The defensive system is automatically activated to keep the feelings repressed and to avoid or alter the emotionally triggering situation. Over time, this pattern is automatically triggered in any situation that has the potential to elicit the repressed feelings about the original attachment rupture.

Consider the case study presented below.

A young violist in her final year of music studies at a prestigious music conservatorium presented with severe music performance anxiety that manifested in hand tremor and bow shake which in turn affected her control of her instrument, rhythmic precision, and tonal quality. She was soon to audition for places in state and national orchestras and feared that her mental state would preclude success. I recommended that she commence beta blockers immediately for symptom relief of severe manifestations of somatic anxiety while we worked psychodynamically on the underlying causes of her MPA. She commented in one session that when she really wanted something, she believed in her mind that she should have it, and when it didn’t happen, she was devastated. This included “having” a perfect performance. This comment had the flavour of “psychic equivalence” – that inner wishes and outer reality should match. This comment felt pivotal to me, suggesting that (part of) her emotional development had been arrested at a very young age. The other significant feature was her fear of appearing either arrogant (paternal introject) or too needy (maternal introject) – uncomfortable opposites inhabiting the same body – rendering her an observer rather than a participant in all her performances and social interactions. She was avoidantly attached to her father, a poorly attuned, authoritarian figure, and experienced a preoccupied attachment with her mother, who modelled fearfulness of and submissiveness towards her husband which our young violist emulated with all authority figures including her therapist, in front of whom she could barely complete a sentence for the first several weeks of psychotherapy.

In Kenny (2011), I report in detail on a number of musicians with whom I have worked psychotherapeutically over the years. I have concluded that the underlying psychopathology of severe MPA is an attachment rupture in early life that is unresponsive to cognitive behavioral therapies. I invite you to revisit these cases as they are most instructive regarding the complexity and multi-dimensionality of music performance anxiety (MPA). Since then, I have published a number of studies on the central role that attachment quality plays in the etiology of MPA in its most severe form (see, for example, Kenny and Holmes, 2015, 2018; Kenny et al., 2016; Kenny, 2016b). These papers investigate attachment themes in the life history narratives of professional musicians and their relationship with MPA. The underpinning hypothesis is that the performance setting re-triggers unprocessed feelings related to early attachment experiences, especially when traumatic, and that defensive manoeuvres against their re-emergence into consciousness are activated. Idiographic research highlights early relational trauma as a relevant etiological factor in the MPA-symptomatic of anxious musicians.

I have therefore argued for an attachment-informed life-course model rather than a purely symptomatic approach to understanding and treating severe MPA and other intra-personal psychodynamics of performing musicians. Empirical support for this hypothesis is now emerging. In conducting attachment-informed intake assessments, it is important to note the following elements in the history: Presence of psychological vulnerability, behavioral inhibition, and/or sensitizing experiences (Kenny and Osborne, 2006), parental mis/attunement, presence or absence of an internal secure base, the quality of current musical experience, and any re-triggering of attachment trauma in current experiences with music performance. These elements of attachment align with Barlow’s three factors–biological vulnerability (negative affect, behavioral inhibition), psychological vulnerability (lack of an internal secure base, experienced as a sense of unpredictability and uncontrollability, and that one does not have the necessary coping resources), and sensitizing past, current, or triggering experiences, all of which I attempted to capture in the K-MPAI.

A recent study (Wiedemann et al., 2020) exploring the relationship between parenting style, adult attachment type, and MPA using a German translation of the K-MPAI showed significant relationships between overcontrolling and abusive parenting style in both parents and indifference in the mother and higher scores on the K-MPAI. Further, significant main effects of adult attachment on MPA were found for the four attachment prototypes [secure, dismissing (also known as avoidant), preoccupied, anxious] with dismissing (avoidant) attachment styles scoring lowest on MPA and anxious and preoccupied scoring highest on MPA. These findings are consistent with the attachment literature regarding how people respond to situations and relationships in adulthood. Secure and dismissing attachment are associated with higher self-concept than anxious and preoccupied attachment, which in turn is associated with the degree of comorbid generalized and specific anxieties (Shaver et al., 2009).

### 4.4. Comorbidity of MPA with other anxiety disorders

There has been ongoing controversy regarding the question as to whether MPA is a form of social anxiety disorder (SAD) (social phobia) (Barbar et al., 2014b), whether it is frequently comorbid with SAD (Kenny, 2011), or whether SAD is independent of MPA (Wiedemann et al., 2022). This is an important question for theoretical, diagnostic, and therapeutic reasons and deserves serious attention. DSM IV (1994) and DSM IV Tr (2000) presented MPA as a subtype of SAD, but others have questioned this (e.g., Kenny, 2011; Kenny, 2016a) arguing that MPA and SAD differ in significant ways and that SAD may be comorbid with MPA rather than MPA being a subtype of SAD. A study of Australian professional orchestral musicians found that one third of this population met criteria for SAD, with more females demonstrating greater comorbidity, as is the case in the general population. The State–Trait Anxiety Inventory (STAI-T), a measure of more generalized anxiety, the Social Phobia Inventory (SPIN), the Anxiety and Depression Detector (ADD), and younger age were all independent predictors of MPA severity (Kenny et al., 2012). A study of 82 music students, using a German translation of the K-MPAI and the disorder-specific anxiety measures of the DSM 5 (APA, 2013)–including agoraphobia, generalized anxiety disorder (GAD), panic disorder (PD), separation anxiety disorder, specific phobia, SAD, and illness anxiety disorder–found GAD scores to be the best predictor of MPA (Wiedemann et al., 2022). This is not surprising given the high correlations reported between STAI-T and MPA, although the DSM measure is diagnostic of GAD and therefore adds to our clinical understanding of the severity of comorbid anxiety conditions. Of interest in this German study is the finding that those student musicians with pathological anxiety profiles consistently reported clinically significant levels of MPA, while those whose anxiety scores were within the normal range had variable MPA scores, falling within both higher and lower ranges of MPA severity. I would hypothesize that those with normal anxiety, but high MPA met my criteria for focal MPA. Such individuals may simply have a biologically more reactive autonomic nervous system rather than any identified psychopathology. It is this group who may benefit most from beta blockers if their anxiety is mostly somatic (as opposed to cognitive) in nature.

However, before we reach any precipitous conclusions on the matter, consider the concept of the “illusion of mental health based on denial or self-deception” proposed by Cousineau and Shedler (2006), pp. 427-432 who found strong associations between the defensive denial of distress and/or mental health issues and higher physiological reactivity. It is important to complement nomothetic with idiopathic, qualitative (Kenny and Holmes, 2018), narrative, and single case design (Kenny and Holmes, 2015) studies to elucidate the puzzling findings from population studies. Individual level analysis highlights that variability is the rule not the exception. For example, counter-intuitively, a study of skilled musical performance in tertiary level flute players showed individualized patterns of concordance-non-concordance between self-reported anxiety on the STAI-T and K-MPAI, heart rate, and EMG measures (Kenny et al., 2013).

There are several other factors that play a role in the genesis and severity of MPA that require further attention. These include depression, low self-esteem, and somatization.

## 5. Depression

Depression has only recently been considered as a comorbidity in the more severe forms of MPA although it is the case that in all forms of anxiety, both general and MPA, anxiety tends to be more severe in the presence of comorbid depression (Kenny, 2011; Kenny et al., 2012; Kenny and Ackermann, 2015). Indeed, depression also has a complex interaction with reports of performance-related musculoskeletal disorders in orchestral musicians, a topic to which I will return in the section on somatization.

Depression can be both a cause and outcome of unresolved MPA, particularly in the presence of low self-esteem and a fragile personality structure. For example, demonstrating cause, Barbar et al. (2014c) investigated professional Brazilian musicians, reporting that musicians with MPA were 3.87 times more likely to develop depression compared with musicians with low or no MPA. Rates of depression within the music sample were high–24% for the professional and 17% for amateur musicians. Demonstrating outcome, Sickert et al. (2022), using a sample of 295 German musicians of varying professional and educational standards, found that three subscales of the K-MPAI–proximal somatic anxiety and worry about performance (factor 1), worry/dread/negative cognitions/ruminations focused on self/other scrutiny (factor 2), and anxious apprehension (factor 3)–predicted depression using the *Beck Depression Inventory*; low self-esteem using *Rosenberg Self-Esteem Scale* also predicted K-MPAI factor scores, which in turn significantly predicted depression. Music students had significantly lower self-esteem compared with employed professional musicians. This finding could represent a self-selection bias among the professional musicians, i.e., only the most talented and confident progressed to professional status in their music careers. Further, they were older and hence more emotionally mature, with greater coping resources and perhaps less maladaptive perfectionism that is often observed in tertiary music students. Nonetheless, this sample of musicians reported more depression at higher levels compared with the general German population. There have been several calls in the literature to examine more closely the impacts of situational factors (Papageorgi, 2022) and musical pedagogy methods in tertiary music institutions globally (Jimenez, 2016; Carrasco, 2019; Clearman, 2020) that may be contributing to poor mental health, loss of confidence, and high MPA in music students.

## 6. Low self-esteem, “false self,” and pathological accommodation

A young woman studying a combined law and music performance degree presented with concerns about what she described as a debilitating form of stage anxiety. She said that it had been prominent as far back as she could remember and that it had prevented her from truly enjoying the one thing she considered a means of refuge in her life. Recently, this anxiety had begun to affect the outlook she had in all areas in her life … “with each performance breakdown, I feel like I am just a useless piece of carbon with no purpose.” This young woman hated her law degree but felt compelled to continue due to parental pressure and expectation. However, she wanted to be a musician. We subsequently discussed how she took in the commands and expectations from outside, leaving no space within to know her own mind. As the elder child of a working mother and invalid father, she became prematurely adult and presented with a “false self” façade. This entailed a pseudo-sophisticated, excessively polite, and deferential manner. She was always trying to be “good”, to please her parents and meet their expectations, to “make something” of herself, so that she would not “be nothing like her father”. The degree of pathological accommodation to externally imposed expectations left her wondering whose life she was living. She needed to separate and individuate from her enmeshed family, and to “discover her own mind.” Several weeks into therapy, she had a revelatory moment. “Wow! A blank slate … I am not liking it … [she burst into tears] … if I am a blank slate or canvas, I am the painter and the canvas; I am standing here with all the paints not knowing which one to choose.”

I have discussed in my book and elsewhere in case reports about the organization and perception of self that underpins all our reactions to life in general and to stressful situations in particular. I refer the interested reader to these sources for a detailed discussion. Suffice to say here that anxiety, attachment, propensity for negative affect and depression, somatization which is a defense against emotional pain, and self-concept/self-efficacy are intricately interwoven. In our study of orchestral musicians, the Core Self Efficacy measure (CSE) (Judge et al., 2003) was negatively correlated with all other measures of general anxiety, MPA, and depression (Kenny et al., 2012). For example, the correlation between K-MPAI and CSE was-0.771, i.e., the lower CSE, the higher MPA. Core self-efficacy encompasses the constructs of self-esteem, locus of control, generalized self-efficacy, and neuroticism (negative affect) and attempts to encapsulate an individual’s sense of “being-in-the-world” in terms of belief in one’s capacity to manage one’s life and its challenges, while maintaining optimism, and a sense of purpose and meaning, even in the face of (perceived) failure and the exigencies of life. These internalized feelings about oneself develop in the primary relationship with the first caregivers (i.e., attachment quality). Lack of attunement, neglect, criticism, overcontrol, overprotectiveness, high expectation with low support, among other dysfunctions in this relationship leaves the young person with a depleted sense of self, fearful of life and doubtful of her capacity. Self-efficacy is also content specific and partly determined by one’s skills and abilities–for example, one can have high self-efficacy for music performance, but low self-efficacy for mathematics. I worked with one young violinist who struggled with severe MPA when playing technically challenging studies and exercises for her teacher. She told me that she was an “esthetic musician” with great capacity for interpretation and musical nuance (high self-efficacy) and she hoped this would make up for her technical deficits (rationalization and minimization of the need for technical competence/mastery). Denial of the importance of central elements to the achievement of an outcome is not an adaptive response to one’s deficits and will ultimately generate significant MPA in young musicians. There has been increasing interest in self-efficacy research in music performance which promises to be a fruitful direction for future research (Egilmez, 2015; Demet, 2017; Dobos and Piko, 2017; Orejudo et al., 2017; González et al., 2018; Zarza-Alzugaray et al., 2020).

## 7. Somatization

An operatic tenor presented to therapy with globus pharyngeus, described as a relatively common persistent or intermittent non-painful sensation of a lump or foreign body in the throat that can make swallowing difficult (Lee and Kim, 2012). Unfortunately, it has uncertain etiology and tends to be treatment resistant. He had received a thorough workup from an otolaryngologist and no physical findings were noted. He had received a trial of proton pump inhibitors assuming possible underlying gastroesophageal reflux with no improvement. He had attended speech and language therapy and was prescribed anti-depressants to no avail. After this diagnostic and clinical marathon, he was referred to me. In the course of history-taking, I asked him whether he was partnered. He replied, “Yes [bringing his hand to his throat] and she [my voice] is a bitch,” whereupon he began to weep.

An association has been established in general medical populations between frequency of reports of acute and chronic pain and severity of pain and comorbid psychological disorders, especially anxiety and depression. There have been few studies on musicians who, by nature of their profession, experience high levels of performance-related musculoskeletal pain disorder (PRMD). Kenny and Ackermann (2015) examined self-reported frequency and severity of PRMD, depression, social phobia, and MPA using K-MPAI in a cross-sectional survey of 377 professional Australian orchestral musicians. Most (84%) musicians had experienced performance-impairing pain; 50% reported current pain at the time of the study. Females reported more performance-impairing pain and more current pain than males. Cluster analysis indicated a complex relationship between depression and PRMD severity. Three clusters showed the expected linear association (i.e., more depression, more pain), but not causality, which may be bidirectional. Musicians in the fourth cluster denied depression but reported the most severe pain, suggesting a group who somatize their psychological distress in the way demonstrated by our operatic tenor. There was also a strong relationship between PRMD and MPA severity, with higher scores on K-MPAI strongly associated with greater PRMD severity. One possible mechanism to account for these findings is the excess muscle tension experienced by anxious people. Tension and anxiety can be expressed in the striated muscles leading to muscle pain and spasms, tremors, or loss of fine motor control or in the smooth muscles, where anxiety is somatized into the gut resulting in more serious somatic symptoms such as nausea, reflux, cramping, and the urge to urinate and/or defecate.

Of course, PRMDs are not attributable solely to psychological factors. Biomechanical factors such as type and weight of instrument, hours practiced, pattern of practice, carrying one’s instrument, and orchestral musician seating all create occupational hazards in the musician’s workplace, in addition to difficulties with conductors, management, and policies and procedures. Biomechanical considerations apply less to singers who carry their voices within their bodies. However, as the vignette above shows, singers have a very complex relationship with their voices. Contextual factors such as music genre specialization (Papageorgi et al., 2011) and quality and type of pedagogy (Jeong and Ryan, 2022) could also contribute to pain, depression, and MPA in musicians and these factors must be assessed simultaneously with any proposed psychological components thought to contribute to pain reports.

## 8. Future directions

Current research has pointed to the possible redundancy of some items in the 40-item version of the K-MPAI. Zarza-Alzugaray et al. (2015) have proposed that “… studies be conducted on the 40-item version of the inventory to further explore interactions between the factors found in the 26-item version, particularly with the aim of assessing specific vulnerability factors proposed by the model which are specified in the 40-item version of the inventory.” I concur with this suggestion. “Early parental/relational context” while emerging as a factor and showing adequate internal consistency in most studies of the factorial structure of the K-MPAI appears more susceptible to cultural influences than the other factors. This may be due to different parenting styles cross-culturally and other environmental variations that may warrant further investigation and more precise specification. Strongly related to this factor is further examination of the role that self-concept/self-esteem, “false self,” and pathological accommodation may play in the development and severity of MPA. The outcome of all these developmental experiences is self-efficacy, but this should not be treated as a discrete variable, rather as an emergent, malleable part of the self that can be modified if identified early in development.

The field of music performance has lagged behind developments in sport psychology in this regard, specifically in the preparation of young people to manage the stress of public performance that is closely scrutinized and evaluated in competitive environments, often before the young person is mature enough to negotiate the pitfalls of premature exposure to such stressors. In assessing MPA, more attention needs to be focused on matters related to pedagogy (e.g., pathological accommodation can occur in the relationship between teacher and student, as well as parent and child, given the importance that teachers assume in preparing young people for musical careers). Practice routines, consolidating technical mastery of the instrument, performance preparation including the suitability of repertoire for the age and development of the young musician, the avoidance of thrusting young musicians into stressful performance situations for which they may not be adequately prepared, ensuring that the child engages in childhood activities like play, sport, leisure, and other intellectual interests apart from music, are all critical to the development of core self-efficacy that is preventive of psychological disorders, including debilitating MPA. The ideal in child development is prevention. In music, we know that sensitizing experiences (i.e., trauma in music performance) can occur early in the young musician’s performance experience and cast a menacing shadow over all subsequent musical endeavors (Osborne and Kenny, 2008). How such experiences can be minimized or avoided is a worthy question that future researchers could helpfully address.

In addition to the K-MPAI, I have developed three MPA rating scales that may warrant further attention. These are (i) *Performance anxiety in different performance settings rating scale* in which musicians rate the degree of anxiety experienced in nine performance situations, including two non-music performance settings (i.e., oral presentation and a written exam); (ii) A 22-item *Perceived causes of music performance anxiety checklist* derived from therapeutic interviews with anxious musicians; and (iii) *Self-management of music performance anxiety rating scale* in which musicians select from a list of 18 strategies those that they used to manage their MPA and to rank each strategy for perceived effectiveness (Kenny, 2011; Kenny et al., 2012). I have found the use of these checklists both illuminating and therapeutically useful.

Another area that warrants further attention is the role that occupational stress and workplace/music study climate, occupational role concerns, and occupational personal strain play in generating or maintaining unacceptably high levels of MPA, together with a study of the resilience and personal resources and coping strategies [see (iii) above] that mitigate these stressors. For example, there are few occupations that require workers to audition every year to maintain their position as occurs in some performing arts companies. In one early study exploring these factors in operatic chorus artists, results showed that higher scores on personal resources were associated with the higher scores on trait anxiety, suggesting that these resources were used adaptively to manage anxiety. High trait anxiety was also associated with higher personal strain in the work environment (Kenny et al., 2004). Differently configured physical working environments may also affect musician well-being and MPA levels experienced during performance (see, e.g., Kenny et al., 2016). Outcomes of investigations in this area may have significant implications for workplace management practices that could ease the stress of professional musicians (Ackermann et al., 2014).

Performance quality, by virtue of the inherent difficulties in measuring it, is too infrequently included in studies of MPA impacts and outcomes of therapeutic interventions. It is a critical variable for both the researcher and the performer and deserves more attention in the MPA literature (Kenny et al., 2013).

Finally, just as medical practice is becoming increasingly personalized with the development of “precision medicine,” so too should our discipline of psychology become more idiographic (i.e., individualized/personalized) in its research interests (Kenny, 2011). Psychology is now essentially the study of groups and populations, in which distilled results are reported in randomized controlled trials, regression, factor, path, and meta-analyzes. Allport (1955) made the same observation when psychology was still in its formative years. He urged us to take the road less travelled, noting that psychologists should continue to struggle with the “dilemma of uniqueness … [that we need to understand that] each person is an idiom unto himself, an apparent violation of the syntax of the species”. We therefore need to be mindful of the unique quality of anxiety in each individual musician in order to advance our field of study in music performance anxiety.

## Author contributions

The author confirms being the sole contributor of this work and has approved it for publication.

## Conflict of interest

Author DK is Principal, DK Consulting.

## Publisher’s note

All claims expressed in this article are solely those of the authors and do not necessarily represent those of their affiliated organizations, or those of the publisher, the editors and the reviewers. Any product that may be evaluated in this article, or claim that may be made by its manufacturer, is not guaranteed or endorsed by the publisher.
