# Supplementary material for: The Kenny music performance anxiety inventory (K-MPAI): Scale construction, cross-cultural validation, theoretical underpinnings, and diagnostic and therapeutic utility
Source: Front Psychol. 2023 May 26;14:1143359. doi: 10.3389/fpsyg.2023.1143359 (PMC10262052; doi:10.3389/fpsyg.2023.1143359)
Supplement: Supplementary file 1 [file Table_1.doc]

Deze vragenlijst gaat over uw podiumervaring en eventuele klachten. Het is de bedoeling dat u telkens aangeeft in hoeverre een uitspraak op u van toepassing is

|  |  | **Sterk oneens** | |  |  |  | **Sterk mee eens** | |
| --- | --- | --- | --- | --- | --- | --- | --- | --- |
| K_1 | Soms voel ik me depressief zonder te weten waarom. Soms voel ik me depressief zonder te weten waarom | 6 | 5 | 4 | 3 | 2 | 1 | 0 |
| K_2 | Ik vind het makkelijk om anderen te vertrouwen | 6 | 5 | 4 | 3 | 2 | 1 | 0 |
| K_3 | Ik vind het makkelijk om anderen te vertrouwen | 0 | 1 | 2 | 3 | 4 | 5 | 6 |
| K_4 | Ik vind het vaak moeilijk de energie te vinden om dingen te gaan doen | 0 | 1 | 2 | 3 | 4 | 5 | 6 |
| K_5 | Veel piekeren is kenmerkend voor mijn familie | 0 | 1 | 2 | 3 | 4 | 5 | 6 |
| K_6 | Ik heb vaak het gevoel dat het leven me niet veel te bieden heeft | 0 | 1 | 2 | 3 | 4 | 5 | 6 |
| K_7 | Hoe harder ik werk ter voorbereiding op een optreden, des te waarschijnlijker het is dat ik een ernstige fout zal maken | 0 | 1 | 2 | 3 | 4 | 5 | 6 |
| K_8 | Ik vind het moeilijk om afhankelijk van anderen te zijn | 0 | 1 | 2 | 3 | 4 | 5 | 6 |
| K_9 | Mijn ouders waren meestal ontvankelijk voor mijn behoeften | 6 | 5 | 4 | 3 | 2 | 1 | 0 |
| K_10 | Ik weet voorafgaand aan een optreden nooit of ik goed zal presteren………………………………………………………….. | 0 | 1 | 2 | 3 | 4 | 5 | 6 |
| K_11 | Ik heb vaak het gevoel dat ik als persoon niet veel waard ben | 0 | 1 | 2 | 3 | 4 | 5 | 6 |
| K_12 | Tijdens een optreden vraag ik me af of ik het überhaupt wel zal redden …………………………………………………………… | 0 | 1 | 2 | 3 | 4 | 5 | 6 |
| K_13 | Nadenken over een mogelijke beoordeling van mijn optreden heeft een negatief effect op mijn prestaties | 0 | 1 | 2 | 3 | 4 | 5 | 6 |
| K_14 | Zelfs tijdens de meest stressvolle optredens, heb ik het vertrouwen dat ik goed zal presteren | 0 | 1 | 2 | 3 | 4 | 5 | 6 |
| K_15 | Ik ben vaak bezorgd over een negatieve reactie van het publiek | 0 | 1 | 2 | 3 | 4 | 5 | 6 |
| K_16 | Soms voel ik me angstig zonder dat daar een bijzondere reden voor is …………………………………………………..………….. | 0 | 1 | 2 | 3 | 4 | 5 | 6 |
| K_17 | Vanaf het begin van mijn muzikale studies herinner ik me dat ik angstig was om op te treden | 6 | 5 | 4 | 3 | 2 | 1 | 0 |
| K_18 | Ik maak me zorgen dat één slecht optreden mijn carrière zal ruïneren | 0 | 1 | 2 | 3 | 4 | 5 | 6 |
| K_19 | Mijn ouders luisterden bijna altijd naar mij | 0 | 1 | 2 | 3 | 4 | 5 | 6 |
| K_20 | Belangrijke kansen om op te treden laat ik schieten vanwege angst | 0 | 1 | 2 | 3 | 4 | 5 | 6 |
| K_21 | Als kind voelde ik me vaak droevig | 0 | 1 | 2 | 3 | 4 | 5 | 6 |
| K_22 | Vaak bereid ik me voor op een optreden met een gevoel van angst en naderend onheil …………………………………..…………..... | 0 | 1 | 2 | 3 | 4 | 5 | 6 |
| K_23 | Ik heb vaak het gevoel dat ik niets heb om naar uit te kijken | 6 | 5 | 4 | 3 | 2 | 1 | 0 |
| K_24 | Mijn ouders moedigden me aan om nieuwe dingen te proberen due to anxiety | 0 | 1 | 2 | 3 | 4 | 5 | 6 |
| K_25 | Ik maak me voorafgaand aan een optreden zoveel zorgen, dat ik niet kan slapen…………………………………………..………..... | 0 | 1 | 2 | 3 | 4 | 5 | 6 |
| K_26 | ijn geheugen is meestal zeer betrouwbaar………………….. | 0 | 1 | 2 | 3 | 4 | 5 | 6 |

©Kenny, D.T. (2004). *Kenny Music Performance Anxiety Inventor-Revised* (K-MPAI) translated by Esther van Femma
