# Supplementary material for: The Kenny music performance anxiety inventory (K-MPAI): Scale construction, cross-cultural validation, theoretical underpinnings, and diagnostic and therapeutic utility
Source: Front Psychol. 2023 May 26;14:1143359. doi: 10.3389/fpsyg.2023.1143359 (PMC10262052; doi:10.3389/fpsyg.2023.1143359)
Supplement: Supplementary file 2 [file Data_Sheet_1.zip › K-MPAI_Bahasa Indonesian translation.pdf]

Dibawah ini adalah beberapa pernyataan tentang apa yang anda rasakan secara umum dan apa yang anda rasakan **sebelum atau selama pertunjukan**. Lingkarilah salah satu nomor untuk menunjukkan seberapa besar anda setuju atau tidak setuju dengan tiap-tiap pernyataan tersebut.

		**Sangat Tidak Setuju**						**Sangat Setuju**
K_1	Saya secara umum merasa mengendalikan hidup saya	6	5	4	3	2	1	0
K_2	Saya merasa mudah mempercayai orang lain	6	5	4	3	2	1	0
K_3	Kadangkala saya merasa tertekan tanpa mengetahui sebabnya	0	1	2	3	4	5	6
K_4	Saya sering merasa kesulitan untuk membangkitkan semangat untuk melakukan sesuatu........	0	1	2	3	4	5	6
K_5	Kecemasan yang berlebihan adalah karakteristik keluarga saya	0	1	2	3	4	5	6
K_6	Saya sering merasa bahwa hidup tidak banyak memberikan pilihan kepada saya	0	1	2	3	4	5	6
K_7	Bahkan jika saya bekerja keras dalam mempersiapkan sebuah pertunjukan, saya mungkin melakukan kesalahan	0	1	2	3	4	5	6
K_8	Saya tidak mudah tergantung pada orang lain	0	1	2	3	4	5	6
K_9	Orang tua saya kebanyakan merespon kebutuhan-kebutuhan saya	6	5	4	3	2	1	0
K_10	Sebelum atau selama pertunjukan, saya merasakan semacam kepanikan	0	1	2	3	4	5	6
K_11	Saya tidak pernah tahu sebelum sebuah konser apakah saya akan tampil dengan baik	0	1	2	3	4	5	6
K_12	Sebelum atau selama pertunjukan, saya merasa mulut saya kering	0	1	2	3	4	5	6
K_13	Saya sering merasa bahwa saya bukanlah siapa-siapa	0	1	2	3	4	5	6
K_14	Selama pertunjukan saya memikirkan apakah saya akan melewatinya	0	1	2	3	4	5	6
K_15	Memikirkan tentang evaluasi saya bisa merasa terganggu dengan penampilan saya	0	1	2	3	4	5	6
K_16	Sebelum atau selama pertunjukan, saya merasa mual atau pusing atau perut yang teraduk	0	1	2	3	4	5	6
K_17	Bahkan di dalam situasi-situasi pertunjukan yang paling menegangkan, saya yakin bahwa saya akan tampil dengan baik	6	5	4	3	2	1	0
K_18	Saya sering merasa peduli dengan reaksi negatif dari penonton	0	1	2	3	4	5	6
K_19	Kadangkala saya merasa cemas tanpa alasan yang jelas	0	1	2	3	4	5	6
K_20	Sejak awal saya belajar musik, saya ingat bahwa saya merasa cemas sebelum tampil	0	1	2	3	4	5	6

BILITON TRANSLATION CENTRE
SURABAYA

		Sangat Tidak Setuju						Sangat Setuju
K_21	Saya khawatir bahwa satu kali penampilan yang buruk dapat menghancurkan karir saya	0	1	2	3	4	5	6
K_22	Sebelum atau selama pertunjukan, saya mengalami dada yang berdegup kencang karena debaran jantung yang meningkat	0	1	2	3	4	5	6
K_23	Orang tua saya hampir selalu mendengarkan saya	6	5	4	3	2	1	0
K_24	Saya meninggalkan peluang-peluang penampilan yang layak	0	1	2	3	4	5	6
K_25	Setelah pertunjukan, saya cemas apakah saya bermain dengan cukup bagus	0	1	2	3	4	5	6
K_26	Kecemasan dan kekhawatiran saya tentang penampilan saya mengganggu fokus dan konsentrasi saya	0	1	2	3	4	5	6
K_27	Sebagai seorang anak, saya sering merasa sedih	0	1	2	3	4	5	6
K_28	Saya sering bersiap untuk sebuah konser dengan perasaan ngeri seolah bencana segera terjadi	0	1	2	3	4	5	6
K_29	Salah satu atau kedua orang tua saya memiliki kecemasan yang berlebihan	0	1	2	3	4	5	6
K_30	Sebelum atau selama pertunjukan, ketegangan otot saya meningkat	0	1	2	3	4	5	6
K_31	Saya sering merasa bahwa saya tidak memiliki sesuatu untuk dinantikan	0	1	2	3	4	5	6
K_32	Setelah pertunjukan, saya mengulangnya kembali di kepala saya berulang-ulang	0	1	2	3	4	5	6
K_33	Orang tua saya mendorong saya untuk mencoba hal-hal baru	6	5	4	3	2	1	0
K_34	Saya sangat merasa cemas sebelum sebuah pertunjukan, saya tidak bisa tidur	0	1	2	3	4	5	6
K_35	Ketika sedang tampil tanpa diiringi musik, ingatan saya dapat diandalkan	6	5	4	3	2	1	0
K_36	Sebelum atau selama pertunjukan, saya merasa bergetar atau gemetar atau tremor	0	1	2	3	4	5	6
K_37	Saya merasa percaya diri bermain menurut ingatan saya	6	5	4	3	2	1	0
K_38	Saya merasa cemas saat sedang diperhatikan oleh orang lain	0	1	2	3	4	5	6
K_39	Saya mencemaskan penilaian saya sendiri tentang bagaimanakah saya akan tampil	0	1	2	3	4	5	6
K_40	Saya tetap berkomitmen untuk tampil meskipun itu membuat saya merasa sangat cemas	0	1	2	3	4	5	6

© Kenny, D.T. (2009), Inventaris Kecemasan Pertunjukan Musik Kenny – Revisi (K-MPAI-R)

BILITON TRANSLATION CENTRE
SURABAYA
BILITON 45
