# Supplementary material for: The Kenny music performance anxiety inventory (K-MPAI): Scale construction, cross-cultural validation, theoretical underpinnings, and diagnostic and therapeutic utility
Source: Front Psychol. 2023 May 26;14:1143359. doi: 10.3389/fpsyg.2023.1143359 (PMC10262052; doi:10.3389/fpsyg.2023.1143359)
Supplement: Supplementary file 2 [file Data_Sheet_1.zip › K-MPAI_Chinese translation.pdf]

下文中是一些关于你的总体感受，以及在**演出之前或演出期间**感受的陈述。请圈出一个数字说明您对每项陈述的同意或不同意程度。

		**非 常 不同意**						**非常 同意**
K_1	我通常感觉自己可以控制自己的生活	6	5	4	3	2	1	0
K_2	我发现自己很容易信任他人	6	5	4	3	2	1	0
K_3	有时我不知为何会感到情绪低落 …… …………….................	0	1	2	3	4	5	6
K_4	我经常发现很难精力充沛地做事 ………………………...........	0	1	2	3	4	5	6
K_5	过度担忧是我家人的特点	0	1	2	3	4	5	6
K_6	我经常感到生活给予我的不多...........................	0	1	2	3	4	5	6
K_7	即使我努力为表演做准备，还是有可能会犯错 ……………………...	0	1	2	3	4	5	6
K_8	我发现很难依赖他人	0	1	2	3	4	5	6
K_9	我的父母大多时候对我的需求做出回应	6	5	4	3	2	1	0
K_10	在演出前或演出期间，我会有类似于恐慌的感觉……………….	0	1	2	3	4	5	6
K_11	在音乐会之前，我永远不知道我是否会表演得很好	0	1	2	3	4	5	6
K_12	在演出前或演出期间，我有口干的体验…………………	0	1	2	3	4	5	6
K_13	我经常感到作为一个人没有太多的价值	0	1	2	3	4	5	6
K_14	在演出期间，我发现自己一直在思考是否能熬过去..............	0	1	2	3	4	5	6
K_15	一想到评估，我的表现就可能会受到干扰.................................	0	1	2	3	4	5	6
K_16	在演出前或演出期间，我感到恶心、晕眩或胃部翻腾……….	0	1	2	3	4	5	6
K_17	即使在压力最大的演出情况下，我也自信于我会表现出色.........	6	5	4	3	2	1	0
K_18	我经常会担心来自观众们的负面反应....................................	0	1	2	3	4	5	6
K_19	有时我会毫无缘由地感到焦虑	0	1	2	3	4	5	6
K_20	从我早期的音乐学习开始，我记得我一直对表演感到焦虑........	0	1	2	3	4	5	6

		非常<br>不同意						非常<br>同意
K_21	我担心一次糟糕的表演可能会毁掉我的职业生涯	0	1	2	3	4	5	6
K_22	在表演前或表演期间，我的心率加快，就像在我胸口处撞击一样………………………………………………………….......	0	1	2	3	4	5	6
K_23	我的父母几乎总是听我的.............................	6	5	4	3	2	1	0
K_24	我会放弃有价值的表演机会	0	1	2	3	4	5	6
K_25	演出结束后，我会担心自己是否表演得足够好……….………….....	0	1	2	3	4	5	6
K_26	我对演出的担忧和紧张会干扰到我的注意力和专注力..	0	1	2	3	4	5	6
K_27	当我还是孩子时，我经常感到悲伤.................................	0	1	2	3	4	5	6
K_28	我经常带着恐惧感和即将到来的灾难感为音乐会做准备………...	0	1	2	3	4	5	6
K_29	我父母中的一方或双方曾经过度焦虑…………….	0	1	2	3	4	5	6
K_30	在表演前或表演期间，我的肌肉紧张加剧了………………...	0	1	2	3	4	5	6
K_31	我经常觉得自己没有什么可期待的....................	0	1	2	3	4	5	6
K_32	表演结束后，我会在脑海中一遍又一遍地回放...	0	1	2	3	4	5	6
K_33	我的父母鼓励过我尝试新的事物.........................	6	5	4	3	2	1	0
K_34	在演出前我非常担忧，无法入睡............	0	1	2	3	4	5	6
K_35	在没有音乐的情况下表演时，我的记忆是可靠的……..	6	5	4	3	2	1	0
K_36	在演出前或演出期间，我感到颤抖或发抖……………...	0	1	2	3	4	5	6
K_37	我有自信凭记忆演奏/演唱........................	6	5	4	3	2	1	0
K_38	我担心被其他人审视 ……………	0	1	2	3	4	5	6
K_39	我担心自己对自己如何表演的判断……	0	1	2	3	4	5	6
K_40	我仍然致力于表演，尽管这会让我非常焦虑…………………..…	0	1	2	3	4	5	6

©肯尼, D.T. (2009). *肯尼音乐表演焦虑发明家-修订版* (K-MPAI-R)

K-MPAI© (肯尼, 2009, 2011) FACTORS	得分	%
1. **近端躯体焦虑和对表演的担忧**		
K_10 在演出前或演出期间，我会有类似于恐慌的感觉		
K_12 在演出前或演出期间，我会有口干的体验		
K_14 在演出期间，我发现自己一直在思考是否能熬过去		
K_16 在演出前或演出期间，我感到恶心、晕眩或胃部翻腾		
K_22 在表演前或表演期间，我的心率加快，如胸部剧烈跳动		
K_26 我对演出的担忧和紧张会干扰到我的注意力和专注力		
K_28我经常带着恐惧感和即将到来的灾难感为音乐会做准备		
K_30 在表演前或表演期间，我的肌肉紧张加剧了		
K_34在演出前我非常担忧，无法入睡		
K_36 在演出前或演出期间，我感到颤抖或发抖		
K_40 我仍然致力于表演，尽管这会让我非常焦虑		
**总计/66**		
**2. 担心/恐惧（消极认知）专注于自我/他人的审视**		
K_7 即使我努力为表演做准备，还是有可能会犯错		
K_15 一想到评估，我的表现就可能会受到干扰		
K_18 我经常会担心来自观众们的负面反应		
K_21我担心一次糟糕的表演可能会毁掉我的职业生涯		
K_25 演出结束后，我会担心自己是否表演得足够好		
K_32 表演结束后，我会在脑海中一遍又一遍地回放		
K_38我担心被其他人审视		
K_39我担心自己对自己会如何表演的判断		
**总计/48**		
**3. 抑郁/绝望（心理脆弱性）**		
K_1 我通常感觉自己可以控制自己的生活		
K_2 我发现自己很容易信任他人		
K_3 有时我不知为何会感到情绪低落		
K_4 我经常发现很难集中精力做事		
K_6 我经常感到生活给予我的不多		
K_8 我发现很难依赖他人		
K_13 我经常感到作为一个人没有太多的价值		
K_31我经常觉得自己没有什么可期待的		
**总计/48**		
**4. 父母的同理心**		
K_9 我的父母大多时候对我的需求作出回应		
K_23 我的父母几乎总是听我的		
K_27 当我还是孩子时，我经常感到悲伤		
K_33 我的父母鼓励过我尝试新的事物		
**总计/24**		
**5. 记忆**		
K_35 在没有音乐的情况下表演时，我的记忆是可靠的		
K_37 我有自信凭记忆演奏/演唱		

**总计/12**		
6. 焦虑的代际传递		
K_5 过度担忧是我家人的特点		
*K_19*有时我会毫无缘由地感到焦虑		
K_29 我父母中的一方或双方曾经过度焦虑		
**总计/18**		
7. 焦虑恐惧		
K_11 在音乐会之前，我永远不知道我是否会表演得很好		
K_17 即使在压力最大的演出情况下，我也自信于我会表现出色		
K_24 由于焦虑，我会放弃有价值的表演机会		
**TOTAL/18**		
8. 生理脆弱性		
K_20 从我早期的音乐学习开始，我记得我一直对表演感到焦虑 **总计/6**		
**综合总计/240**		
