# Supplementary material for: The Kenny music performance anxiety inventory (K-MPAI): Scale construction, cross-cultural validation, theoretical underpinnings, and diagnostic and therapeutic utility
Source: Front Psychol. 2023 May 26;14:1143359. doi: 10.3389/fpsyg.2023.1143359 (PMC10262052; doi:10.3389/fpsyg.2023.1143359)
Supplement: Supplementary file 2 [file Data_Sheet_1.zip › K-MPAI_Croatian translation.pdf]

KMPA-I SKALA ANKSIOZNOSTI PRILIKOM JAVNOG GLAZBENOG NASTUPANJA

Pročitajte slijedeće tvrdnje o tome kako se **općenito osjećate i kako se osjećate prije ili tijekom javnog nastupa.** Zaokružite broj koji najbolje opisuje u kojoj mjeri se slažete ili ne slažete sa svakom od navedenih tvrdnji.

	**U potpunosti se ne slažem**						**U potpunosti se slažem**
K_1 Općenito osjećam da imam kontrolu nad vlastitim životom.........	6	5	4	3	2	1	0
K_2 Vjerovati drugima je za mene jednostavno.................................	6	5	4	3	2	1	0
K_3 Ponekad se osjećam depresivno bez posebnog razloga...............	0	1	2	3	4	5	6
K_4 Često nemam dovoljno energije da nešto uradim........................	0	1	2	3	4	5	6
K_5 Pretjerna zabrinutost je karakteristika moje obitelji....................	0	1	2	3	4	5	6
K_6 Često osjećam da mi život nema mnogo za ponuditi....................	0	1	2	3	4	5	6
K_7 Čak i kada uložim veliki napor u pripreme za nastup, velika je vjerojatnoća da ću pogriješiti...	0	1	2	3	4	5	6
K_8 Teško mi je ovisiti o drugima...	0	1	2	3	4	5	6
K_9 Moji roditelji su uglavnom bili osjetljivi na moje potrebe..........	6	5	4	3	2	1	0
K_10 Prije ili tijekom nastupa me obuzme osjećaj sličan panici.........	0	1	2	3	4	5	6
K_11 Prije koncerta nikad ne znam hoću li dobro nastupiti.................	0	1	2	3	4	5	6
K_12 Prije ili tijekom koncerta imam osjećaj suhoće u ustima...........	0	1	2	3	4	5	6
K_13 Često osjećam da kao osoba nemam veliku vrijednost...............	0	1	2	3	4	5	6
K_14 Tijekom nastupa razmišljam o tome hoću li uopće uspjeti doći do kraja...	0	1	2	3	4	5	6
K_15 Misli o ocjeni ometaju moj nastup..	0	1	2	3	4	5	6
K_16 Prije ili tijekom nastupa osjećam mučninu, slabost i nelagodu u trbuhu..	6	5	4	3	2	1	0
K_17 Čak i u najstresnijim situacijama sam uvjeren/a da ću dobro nastupiti..	6	5	4	3	2	1	0
K_18 Često me brinu negativne reakcije publike................................	0	1	2	3	4	5	6
K_19 Ponekad se osjećam anksiozno bez posebnog razloga...............	0	1	2	3	4	5	6

K_20 Od početka svog glazbenog obrazovanja se sjećam da

	**U potpunosti se ne slažem**					**U potpunosti se slažem**
sam se pred nastup osjećao/la tjeskobno.. 0	1	2	3	4	5	6

K_21 Zabrinut/a sam da bi jedan loš nastup mogao

upropastiti moju karijeru.. 0 1 2 3 4 5 6

K_22 Prije ili tijekom nastupa moje srce ubrzano kuca,

kao da bi htjelo iskočiti iz grudi... 0 1 2 3 4 5 6

K_23 Moji roditelji su me gotovo uvijek htjeli saslušati..................... 6 5 4 3 2 1 0

K_24 Odustajem od značajnih prilika za javne nastupe...................... 0 1 2 3 4 5 6

K_25 Nakon nastupa brinem jesam li dovoljno dobro svirao/la........... 0 1 2 3 4 5 6

K_26 Briga i nervoza oko nastupa ometaju moju koncentaciju............ 0 1 2 3 4 5 6

K_27 U djetinjstvu sam često bio/bila tužan/tužna.............................. 0 1 2 3 4 5 6

K_28 Često se pripremam za koncert s osjećajem strepnje

i predviđam katastrofu... 0 1 2 3 4 5 6

K_29 Jedan ili oba moja roditelja su bili pretjerano anksiozni........... 0 1 2 3 4 5 6

K_30 Prije ili tijekom nastupa osjećam povećanu napetost mišića...... 0 1 2 3 4 5 6

K_31 Često osjećam da se nemam čemu radovati............................... 0 1 2 3 4 5 6

K_32 Nakon nastupa si cijeli događaj neprestalno

prevrćem po glavi.. 0 1 2 3 4 5 6

K_33 Moji roditelji su me podsticali na to da se okušam

u različitim aktivnostima.. 6 5 4 3 2 1 0

K_34 Prije nastupa sam toliko zabrinut/a da ne mogu spavati............ 0 1 2 3 4 5 6

K_35 Kada sviram bez nota, moje pamćenje je pouzdano.................. 6 5 4 3 2 1 0

K_36 Prije ili tijekom nastupa se tresem ili osjećam drhtavicu........... 0 1 2 3 4 5 6

K_37 Kada sviram napamet osjećam se sigurno................................. 6 5 4 3 2 1 0

K_38 Zabrinut/a sam da će me ostali pažljivo promatrati................... 0 1 2 3 4 5 6

K_39 Zabrinut/a sam zbog prerane samoprocjene svog

budućeg nastupa.. 0 1 2 3 4 5 6

K_40 Ostajem predan/a javnom nastupanju i unatoč tome što

izaziva snažan osjećaj tjeskobe... 0 1 2 3 4 5 6

©Kenny, D.T. (2009). *Kenny Music Performance Anxiety Inventory-Revised* (K-MPAI-R)
