# Supplementary material for: The Kenny music performance anxiety inventory (K-MPAI): Scale construction, cross-cultural validation, theoretical underpinnings, and diagnostic and therapeutic utility
Source: Front Psychol. 2023 May 26;14:1143359. doi: 10.3389/fpsyg.2023.1143359 (PMC10262052; doi:10.3389/fpsyg.2023.1143359)
Supplement: Supplementary file 2 [file Data_Sheet_1.zip › K-MPAI_Czech translation.pdf]

KENNY ŠKÁLA ÚZKOSTI PŘI VEŘEJNÉM HUDEBNÍM VYSTUPOVÁNÍ

Níže je uvedeno několik tvrzení, o tom jak se **obecně cítíte a jak se cítíte před nebo během vystoupení.** Zakroužkujte prosím číslo, které nejlépe vyjadřuje, v jaké míře souhlasíte či nesouhlasíte s každým uvedeným výrokem.

	**Naprosto nesouhlasím**					**Naprosto souhlasím**	
K_1 Obecně cítím, že mám kontrolu nad svým životem...........	6	5	4	3	2	1	0
K_2 Je pro mě snadné důvěřovat ostatním...............................	6	5	4	3	2	1	0
K_3 Občas se cítím depresivně, aniž bych věděl/a proč...........	0	1	2	3	4	5	6
K_4 Často mi příjde obtížné posbírat energii na aktivity..........	0	1	2	3	4	5	6
K_5 Moje rodina si obecně dělá příliš velké starosti................	0	1	2	3	4	5	6
K_6 Občas cítím, že mi život nemá co nabídnout.....................	0	1	2	3	4	5	6
K_7 I když se na vystoupení hodně připravuji, stává se mi, že dělám chyby..	0	1	2	3	4	5	6
K_8 Jsem nerad závislý/á na jiných lidech...............................	0	1	2	3	4	5	6
K_9 Moji rodiče většinou rozuměli mým potřebám.................	6	5	4	3	2	1	0
K_10 Před nebo během vystoupení mě zaplaví pocit podobný panice..	0	1	2	3	4	5	6
K_11 Před koncertem nikdy nevím, jestli zahraji dobře...........	0	1	2	3	4	5	6
K_12 Před nebo během vystoupení cítím sucho v ústech...........	0	1	2	3	4	5	6
K_13 Často cítím, že jako osoba nemám velkou hodnotu.........	0	1	2	3	4	5	6
K_14 Během vystoupení přemýšlím o tom, jestli to vůbec zvládnu dohrát do konce...	0	1	2	3	4	5	6
K_15 Myšlenky o hodnocení, které dostanu, mě obecně ruší při vystoupení..	0	1	2	3	4	5	6
K_16 Před nebo během vystoupení mám pocit na omdlení, cítím nevolnost nebo zvedání žaludku......................................	0	1	2	3	4	5	6
K_17 I v nejvíce stresujících situacích jsem si jistý/á, že zahraji dobře..	6	5	4	3	2	1	0
K_18 Často se obávám negativních reakcí publika..................	0	1	2	3	4	5	6
K_19 Občas se cítím úzkostně i bez konkrétního důvodu.........	0	1	2	3	4	5	6

	Naprosto nesouhlasím						Naprosto souhlasím
K_20 Od začátku svého hudebního vzdělávání si pamatuji, že jsem měl/a z vystupování úzkost..	0	1	2	3	4	5	6
K_21 Obávám se, že by jedno špatné vystoupení mohlo zničit mou kariéru..	0	1	2	3	4	5	6
K_22 Před nebo během vystoupení se mi zrychlí tep, jako by mi bušilo v hrudi..	0	1	2	3	4	5	6
K_23 Moji rodiče mi téměř vždy naslouchali..........................	6	5	4	3	2	1	0
K_24 Vzdávám se významných koncertních příležitostí..........	0	1	2	3	4	5	6
K_25 Po vystoupení mám obavy z toho, jestli jsem zahrál/a dostatečně dobře..	0	1	2	3	4	5	6
K_26 Obavy a nervozita z vystoupení narušují moji koncentraci...	0	1	2	3	4	5	6
K_27 V dětství jsem často byl/a smutný/á...............................	0	1	2	3	4	5	6
K_28 Často se připravuji na koncert s pocitem hrůzy a blížící se katastrofy..	0	1	2	3	4	5	6
K_29 Jeden nebo oba z mých rodičů byly příliš úzkostliví.......	0	1	2	3	4	5	6
K_30 Před nebo během vystoupení cítím zvýšené svalové napětí...	0	1	2	3	4	5	6
K_31 Často cítím, že se nemám na co těšit.............................	0	1	2	3	4	5	6
K_32 Po vystoupení si ho pořád dokola přehrávám v hlavě......	0	1	2	3	4	5	6
K_33 Rodiče mě podporovali, abych zkoušela nové věci.........	6	5	4	3	2	1	0
K_34 Před vystoupením kvůli obavám nemůžu spát................	0	1	2	3	4	5	6
K_35 Když hraji bez not, moje paměť je spolehlivá................	6	5	4	3	2	1	0
K_36 Před nebo během vystoupení se chvěji, třesu nebo mám tremor..	0	1	2	3	4	5	6
K_37 Při hře zpaměti se cítím jistě..	6	5	4	3	2	1	0
K_38 Obávám se, že by mě ostatní mohli podrobně zkoumat..	0	1	2	3	4	5	6
K_39 Obávám se odhadovat svůj budoucí výkon.....................	0	1	2	3	4	5	6
K_40 Jsem pořád odhodlaný/á věřejně vystupovat, i přestože mi to způsobuje velkou úzkost.................................	0	1	2	3	4	5	6

©Kenny, D.T. (2009). *Kenny Music Performance Anxiety Inventory-Revised* (K-MPAI-R)

View publication stats
