# Supplementary material for: The Kenny music performance anxiety inventory (K-MPAI): Scale construction, cross-cultural validation, theoretical underpinnings, and diagnostic and therapeutic utility
Source: Front Psychol. 2023 May 26;14:1143359. doi: 10.3389/fpsyg.2023.1143359 (PMC10262052; doi:10.3389/fpsyg.2023.1143359)
Supplement: Supplementary file 2 [file Data_Sheet_1.zip › K-MPAI_English.pdf]

Below are some statements about how you feel generally and how you feel **before or during a performance**. Please circle one number to indicate how much you agree or disagree with each statement.

		Strongly Disagree						Strongly Agree
K_1	I generally feel in control of my life...	6	5	4	3	2	1	0
K_2	I find it easy to trust others..	6	5	4	3	2	1	0
K_3	Sometimes I feel depressed without knowing why	0	1	2	3	4	5	6
K_4	I often find it difficult to work up the energy to do things...........	0	1	2	3	4	5	6
K_5	Excessive worrying is a characteristic of my family	0	1	2	3	4	5	6
K_6	I often feel that life has not much to offer me...........................	0	1	2	3	4	5	6
K_7	Even if I work hard in preparation for a performance, I am likely to make mistakes	0	1	2	3	4	5	6
K_8	I find it difficult to depend on others	0	1	2	3	4	5	6
K_9	My parents were mostly responsive to my needs	6	5	4	3	2	1	0
K_10	Prior to, or during a performance, I get feelings akin to panic…………………………………………………………….	0	1	2	3	4	5	6
K_11	I never know before a concert whether I will perform well	0	1	2	3	4	5	6
K_12	Prior to, or during a performance, I experience dry mouth…………………………………………………………	0	1	2	3	4	5	6
K_13	I often feel that I am not worth much as a person.....................	0	1	2	3	4	5	6
K_14	During a performance I find myself thinking about whether I'll even get through it	0	1	2	3	4	5	6
K_15	Thinking about the evaluation I may get interferes with my performance	0	1	2	3	4	5	6
K_16	Prior to, or during a performance, I feel sick or faint or have a churning in my stomach…………………………………………..	0	1	2	3	4	5	6
K_17	Even in the most stressful performance situations, I am confident that I will perform well...	6	5	4	3	2	1	0
K_18	I am often concerned about a negative reaction from the audience..	0	1	2	3	4	5	6
K_19	Sometimes I feel anxious for no particular reason....................	0	1	2	3	4	5	6
K_20	From early in my music studies, I remember being anxious about performing	0	1	2	3	4	5	6

		Strongly disagree						Strongly Agree
K_21	I worry that one bad performance may ruin my career	0	1	2	3	4	5	6
K_22	Prior to, or during a performance, I experience increased heart rate like pounding in my chest…………………………....	0	1	2	3	4	5	6
K_23	My parents almost always listened to me	6	5	4	3	2	1	0
K_24	I give up worthwhile performance opportunities	0	1	2	3	4	5	6
K_25	After the performance, I worry about whether I played well enough……………………………………………….……….....	0	1	2	3	4	5	6
K_26	My worry and nervousness about my performance interferes with my focus and concentration………………………………..	0	1	2	3	4	5	6
K_27	As a child, I often felt sad	0	1	2	3	4	5	6
K_28	I often prepare for a concert with a sense of dread and impending disaster……………………………………………....	0	1	2	3	4	5	6
K_29	One or both of my parents were overly anxious……………….	0	1	2	3	4	5	6
K_30	Prior to, or during a performance, I have increased muscle tension…………………………………………………………..	0	1	2	3	4	5	6
K_31	I often feel that I have nothing to look forward to	0	1	2	3	4	5	6
K_32	After the performance, I replay it in my mind over and over...	0	1	2	3	4	5	6
K_33	My parents encouraged me to try new things	6	5	4	3	2	1	0
K_34	I worry so much before a performance, I cannot sleep............	0	1	2	3	4	5	6
K_35	When performing without music, my memory is reliable……..	6	5	4	3	2	1	0
K_36	Prior to, or during a performance, I experience shaking or trembling or tremor……………………………………………....	0	1	2	3	4	5	6
K_37	I am confident playing from memory......................................	6	5	4	3	2	1	0
K_38	I am concerned about being scrutinized by others ………….	0	1	2	3	4	5	6
K_39	I am concerned about my own judgement of how I will perform………………………………………………………..….	0	1	2	3	4	5	6
K_40	I remain committed to performing even though it causes me great anxiety……………………………………………………..….	0	1	2	3	4	5	6

©Kenny, D.T. (2009). *Kenny Music Performance Anxiety Inventor-Revised* (K-MPAI-R)

K-MPAI© (Kenny, 2009, 2011) FACTORS	SCORE	%
1. ***Proximal somatic anxiety and worry about performance***		
K_10 Prior to, or during a performance, I get feelings akin to panic		
K_12 Prior to, or during a performance, I experience dry mouth		
K_14 During a performance I find myself thinking about whether I'll even get through it		
K_16 Prior to, or during a performance, I feel sick or faint or have a churning in my stomach		
K_22 Prior to, or during a performance, I experience increased heart rate like pounding in my chest		
K_26 My worry and nervousness about my performance interferes with my focus and concentration		
K_28 I often prepare for a concert with a sense of dread and impending disaster		
K_30 Prior to, or during a performance, I have increased muscle tension		
K_34 I worry so much before a performance, I cannot sleep		
K_36 Prior to, or during a performance, I experience shaking or trembling or tremor		
K_40 I remain committed to performing even though it causes me significant anxiety		
**TOTAL/66**		
2. Worry/dread (Negative cognitions) focused on self/other scrutiny		
K_7 Even if I work hard in preparation for a performance, I am likely to make mistakes		
K_15 Thinking about the evaluation I may get interferes with my performance		
K_18 I am often concerned about a negative reaction from the audience		
K_21 I worry that one bad performance may ruin my career		
K_25 After the performance, I worry about whether I played well enough		
K_32 After the performance, I replay it in my mind over and over		
K_38 I am concerned about being scrutinized by others		
K_39 I am concerned about my own judgment of how I performed		
**TOTAL/48**		
3. ***Depression/hopelessness (Psychological vulnerability)***		
K_1 I generally feel in control of my life (-)*		
K_2 I find it easy to trust others (-)*		
K_3 Sometimes I feel depressed without knowing why		
K_4 I often find it difficult to work up the energy to do things		
K_6 I often feel that life has not much to offer me		
K_8 I find it difficult to depend on others		
K_13 I often feel that I am not worth much as a person		
K_31 I often feel that I have nothing to look forward to		
**TOTAL/48**		
4. Parental empathy		
K_9 My parents were mostly responsive to my needs (-)*		
K_23 My parents always listened to me (-)*		
K_27 As a child, I often felt sad		
K_33 My parents encouraged me to try new things (-)*		
**TOTAL/24**		
5. Memory		
K_35 When performing without music, my memory is reliable (-)*		
K_37 I am confident playing from memory (-)*		
**TOTAL/12**		
6. Generational transmission of anxiety		
K_5 Excessive worrying is a characteristic of my family		
K_19 Sometimes I feel anxious for no particular reason		
K_29 One or both of my parents were overly anxious		
**TOTAL/18**		
7. Anxious apprehension		
K_11 I never know before a concert whether I will perform well		
K_17 Even in the most stressful performance situations, I am confident that I will perform well (-)*		
K_24 I give up worthwhile performance opportunities due to anxiety		
**TOTAL/18**		
8. Biological vulnerability		
K_20 From early in my music studies, I remember being anxious about performing **TOTAL/6**		
**OVERALL TOTAL/240**		
