# Supplementary material for: The Kenny music performance anxiety inventory (K-MPAI): Scale construction, cross-cultural validation, theoretical underpinnings, and diagnostic and therapeutic utility
Source: Front Psychol. 2023 May 26;14:1143359. doi: 10.3389/fpsyg.2023.1143359 (PMC10262052; doi:10.3389/fpsyg.2023.1143359)
Supplement: Supplementary file 2 [file Data_Sheet_1.zip › K-MPAI_French translation.pdf]

Vous trouverez ci-dessous quelques déclarations sur la façon dont vous vous sentez généralement et sur la façon dont vous vous sentez avant ou pendant une représentation. Veuillez encercler un chiffre pour indiquer dans quelle mesure vous êtes d'accord ou pas d'accord avec chaque énoncé.

		**Pas du tout d'accord**						**Tout à fait d'accord**
K_1	De manière générale je me sens aux commandes de ma vie ..	6	5	4	3	2	1	0
K_2	Je trouve facile de faire confiance aux autres	6	5	4	3	2	1	0
K_3	Parfois je me sens déprimé sans savoir pourquoi	0	1	2	3	4	5	6
K_4	J'ai souvent du mal à trouver l'énergie pour faire des choses ..	0	1	2	3	4	5	6
K_5	S'inquiéter excessivement est une caractéristique de ma famille	0	1	2	3	4	5	6
K_6	J'ai souvent le sentiment que la vie n'a pas grand-chose à m'offrir..	0	1	2	3	4	5	6
K_7	Même si je travaille dur en préparation d'une représentation, je vais probablement faire des fautes	0	1	2	3	4	5	6
K_8	Je trouve difficile de dépendre des autres	0	1	2	3	4	5	6
K_9	La plupart du temps, mes parents ont répondu à mes besoins	6	5	4	3	2	1	0
K_10	Avant ou pendant une représentation, j'ai une sensation semblable à de la panique	0	1	2	3	4	5	6
K_11	Je ne sais jamais avant un concert si je vais bien jouer	0	1	2	3	4	5	6
K_12	Avant ou pendant une représentation, je sens que ma bouche devient sèche.	0	1	2	3	4	5	6
K_13	J'estime souvent que je ne vaux pas grand-chose en tant que personne	0	1	2	3	4	5	6
K_14	Durant une représentation, je me retrouve à penser « est-ce que je vais m'en sortir	0	1	2	3	4	5	6
K_15	Penser à l'évaluation, que je pourrais recevoir, interfère avec ma performance..	0	1	2	3	4	5	6
K_16	Avant ou pendant une représentation, je me sens malade ou faible ou j'ai l'estomac noué	0	1	2	3	4	5	6
K_17	Même dans les situations de représentation les plus stressantes, je suis sûr que je jouerai bien	6	5	4	3	2	1	0
K_18	Je m'inquiète souvent d'une réaction négative du public..........	0	1	2	3	4	5	6
K_19	Parfois je me sens anxieux sans raison particulière	0	1	2	3	4	5	6
K_20	Dès le début de mes études de musique, je me souviens d'avoir été anxieux de jouer	0	1	2	3	4	5	6

		Pas du tout d'accord						Tout à fait d'accord
K_21	Je m'inquiète qu'une mauvaise représentation puisse ruiner ma carrière	0	1	2	3	4	5	6
K_22	Avant ou durant une représentation, je ressens une augmentation de mon rythme cardiaque, comme des battements dans ma poitrine	0	1	2	3	4	5	6
K_23	Mes parents m'ont toujours écouté	6	5	4	3	2	1	0
K_24	Je laisse tomber des possibilités de représentations intéressantes à cause de l'anxiété	0	1	2	3	4	5	6
K_25	Après une représentation, je m'inquiète de savoir si j'ai joué assez bien	0	1	2	3	4	5	6
K_26	Mon inquiétude et ma nervosité à propos de ma prestation interfèrent avec ma focalisation et ma concentration	0	1	2	3	4	5	6
K_27	Enfant, je me sentais souvent triste	0	1	2	3	4	5	6
K_28	Je me prépare souvent pour un concert avec un sentiment de terreur et de désastre imminent.	0	1	2	3	4	5	6
K_29	Un ou mes parents étaient excessivement anxieux	0	1	2	3	4	5	6
K_30	Avant ou pendant une représentation, ma tension musculaire augmente	0	1	2	3	4	5	6
K_31	J'ai souvent l'impression que je n'ai rien pour me réjouir	0	1	2	3	4	5	6
K_32	Après une représentation, je la rejoue dans mon esprit encore et encore	0	1	2	3	4	5	6
K_33	Mes parents m'encourageaient à essayer de nouvelles choses.	6	5	4	3	2	1	0
K_34	Je me fais tellement de soucis avant une représentation que je ne peux pas dormir	0	1	2	3	4	5	6
K_35	Lors des prestations sans partition, ma mémoire est fiable	6	5	4	3	2	1	0
K_36	Avant ou durant une représentation, je ressens des tremblements ou des tressaillements	0	1	2	3	4	5	6
K_37	Je suis confiant quant à ma capacité à jouer de mémoire	6	5	4	3	2	1	0
K_38	J'ai peur d'être minutieusement examiné par d'autres	0	1	2	3	4	5	6
K_39	Je m'inquiète de mon propre jugement sur la façon dont je jouerai	0	1	2	3	4	5	6
K_40	Je continue à m'appliquer à jouer même si cela me cause considérablement d'anxiété	0	1	2	3	4	5	6

©Kenny, D.T. (2009). *Kenny Music Performance Anxiety Inventor-Revised* (K-MPAI-R)
