# Supplementary material for: The Kenny music performance anxiety inventory (K-MPAI): Scale construction, cross-cultural validation, theoretical underpinnings, and diagnostic and therapeutic utility
Source: Front Psychol. 2023 May 26;14:1143359. doi: 10.3389/fpsyg.2023.1143359 (PMC10262052; doi:10.3389/fpsyg.2023.1143359)
Supplement: Supplementary file 2 [file Data_Sheet_1.zip › K-MPAI_German translation.pdf]

Im Folgenden finden Sie einige Aussagen darüber, wie Sie sich allgemein fühlen und wie Sie sich vor oder während einer Vorstellung fühlen. Bitte kreuzen Sie eine Zahl an, um anzugeben, wie viel Sie mit jeder Aussage einverstanden sind oder nicht.

		**Entschieden widersprechen**						**Stimmen sehr zu**
K_1	Im Allgemeinen habe ich das Gefühl mein Leben unter Kontrolle zu haben	6	5	4	3	2	1	0
K_2	Mir fällt es leicht, anderen zu vertrauen	6	5	4	3	2	1	0
K_3	Manchmal fühle ich mich deprimiert, ohne zu wissen warum	0	1	2	3	4	5	6
K_4	Oft fällt es mir schwer, mich zu motivieren, Dinge zu erledigen	0	1	2	3	4	5	6
K_5	Übermässiges Sorgenmachen ist ein charakteristisches Merkmal meiner Familie	0	1	2	3	4	5	6
K_6	Ich habe oft das Gefühl, dass das Leben mir nicht viel zu bieten hat	0	1	2	3	4	5	6
K_7	Selbst wenn ich hart arbeite, um mich auf eine Aufführung vorzubereiten, werde ich wahrscheinlich Fehler machen	0	1	2	3	4	5	6
K_8	Ich finde es schwierig, mich auf andere Menschen zu verlassen	0	1	2	3	4	5	6
K_9	Meine Eltern sind meistens auf meine Bedürfnisse eingegangen	6	5	4	3	2	1	0
K_10	Vor oder während einer Aufführung habe ich panikartige Gefühle	0	1	2	3	4	5	6
K_11	Vor einem Konzert weiss ich nie, ob ich das Stück gut aufführen werde	0	1	2	3	4	5	6
K_12	Vor oder während einer Aufführung habe ich einen trockenen Mund.	0	1	2	3	4	5	6
K_13	Ich habe oft das Gefühl, als Person nicht viel wert zu sein	0	1	2	3	4	5	6
K_14	Während einer Aufführung geht mir durch den Kopf, ob ich überhaupt durch das Stück komme	0	1	2	3	4	5	6
K_15	Der Gedanke an die Beurteilung, die ich bekommen könnte, beeinträchtigt meine Leistung	0	1	2	3	4	5	6
K_16	Vor oder während einer Aufführung fühle ich mich krank oder schwach oder ich habe ein flaues Gefühl im Magen	0	1	2	3	4	5	6
K_17	Sogar in den stressigsten Aufführungssituationen bin ich zuversichtlich, dass ich eine gute Leistung erbringen werde	6	5	4	3	2	1	0
K_18	Ich bin oft über eine negative Reaktion vom Publikum besorgt	0	1	2	3	4	5	6
K_19	Manchmal fühle ich mich ohne erkennbaren Grund beunruhigt	0	1	2	3	4	5	6
K_20	Von meiner frühen musikalischen Ausbildung erinnere ich mich, wegen Aufführungen besorgt gewesen zu sein	0	1	2	3	4	5	6

K_21	Ich mache mir Sorgen, dass eine schlecte Aufführung meine Karriere ruinieren könnte	0	1	2	3	4	5	6
K_22	Vor oder während einer Aufführung erlebe ich eine erhöhte Herzfrequenz wie ein Pochen in meiner Brust	0	1	2	3	4	5	6
K_23	Meine Eltern haben mir immer zugehört	6	5	4	3	2	1	0
K_24	Aus Ängstlichkeit lasse ich lohnenswerte Aufführungsgelegenheiten ausy	0	1	2	3	4	5	6
K_25	Nach der Aufführung mache ich mir Sorgen, ob ich gut genug gespielt habe	0	1	2	3	4	5	6
K_26	Meine Sorge und Nervosität über meine Aufführung beeinträchtigen meinen Fokus und meine Konzentration	0	1	2	3	4	5	6
K_27	Als Kind habe ich mich oft traurig gefühlt	0	1	2	3	4	5	6
K_28	Bei der Vorbereitung auf ein Konzert habe ich oft ein Gefühl von Furcht und einem bevorstehenden Desaster	0	1	2	3	4	5	6
K_29	Eines oder beide meiner Elternteile waren übermässig ängstlich	0	1	2	3	4	5	6
K_30	Vor oder während einer Aufführung habe ich eine erhöhte Muskelspannung	0	1	2	3	4	5	6
K_31	Ich habe oft das Gefühl, dass ich nichts habe, worauf ich mich freuen kann	0	1	2	3	4	5	6
K_32	Nach einer Aufführung spiele ich sie in Gedanken wieder und wieder durch	0	1	2	3	4	5	6
K_33	Meine Eltern haben mich ermutigt, neue Dinge zu probieren	6	5	4	3	2	1	0
K_34	Vor einer Aufführung mache ich mir so viele Sorgen, dass ich nicht schlafen kann	0	1	2	3	4	5	6
K_35	Bei Aufführungen ohne Notenblatt ist mein Gedächtnis zuverlässig	6	5	4	3	2	1	0
K_36	Vor oder nach einer Aufführung leide ich unter Schütteln, Zittern oder Zucken	0	1	2	3	4	5	6
K_37	Ich bin zuversichtlich, wenn ich aus dem Gedächtnis spiele	6	5	4	3	2	1	0
K_38	Ich mache mir Sorgen, von anderen bewertet zu werden	0	1	2	3	4	5	6
K_39	Ich mache mir Sorgen um mein eigenes Urteil über meine Aufführung	0	1	2	3	4	5	6
K_40	Ich bleibe dem Aufführen verbunden, auch wenn es mir erhebliche Angst bereitet	0	1	2	3	4	5	6

©Kenny, D.T. (2009). *Kenny Music Performance Anxiety Inventor-Revised* (K-MPAI-R)
