# Supplementary material for: The Kenny music performance anxiety inventory (K-MPAI): Scale construction, cross-cultural validation, theoretical underpinnings, and diagnostic and therapeutic utility
Source: Front Psychol. 2023 May 26;14:1143359. doi: 10.3389/fpsyg.2023.1143359 (PMC10262052; doi:10.3389/fpsyg.2023.1143359)
Supplement: Supplementary file 2 [file Data_Sheet_1.zip › K-MPAI_Hungarian translation.pdf]

Zenei teljesítményszorongás leltár (K–MPAI)

Az alábbiakban pár állítást olvashat arról, hogy általában hogy érzi magát fellépés **előtt vagy fellépés közben**.

		**Egyáltalán nem értek egyet**						**Teljesen egyetértek**
K_1	Általánosságban véve ura vagyok az életemnek	6	5	4	3	2	1	0
K_2	Könnyen megbízom másokban..	6	5	4	3	2	1	0
K_3	Néha ok nélkül lehangoltnak érzem magam	0	1	2	3	4	5	6
K_4	Gyakran nehezen szedem össze az energiát, hogy megcsináljak dolgokat	0	1	2	3	4	5	6
K_5	A túlzott aggodalmaskodás jellemző a családomra	0	1	2	3	4	5	6
K_6	Gyakran érzem azt, hogy az élettől nem kapok sok mindent....	0	1	2	3	4	5	6
K_7	Még ha sokat is gyakoroltam a fellépés előtt, valószínű, hogy hibákat ejtek..	0	1	2	3	4	5	6
K_8	Nehéz dolog számomra, hogy másoktól függjek	0	1	2	3	4	5	6
K_9	A szüleim többnyire reagáltak a szükségleteimre	6	5	4	3	2	1	0
K_10	Fellépés előtt vagy közben pánikszerű érzéseim vannak.........	0	1	2	3	4	5	6
K_11	Koncert előtt sose tudom, hogy jól fogok-e játszani.................	0	1	2	3	4	5	6
K_12	Koncert előtt vagy közben kiszárad a szám	0	1	2	3	4	5	6
K_13	Gyakran érzem azt, hogy nem vagyok értékes ember.............	0	1	2	3	4	5	6
K_14	Koncert közben gyakran eszembe jut, hogy túljutok-e rajta......	0	1	2	3	4	5	6
K_15	Ha arra gondolok, hogy az előadásomat értékelni fogják, az akadályozza a eljesítményemet	0	1	2	3	4	5	6
K_16	Koncert előtt/közben rosszul érzem magam, erőtlen vagyok és összeszorul a gyomrom..	0	1	2	3	4	5	6
K_17	Még a legstresszesebb előadói helyzetben is biztos vagyok abban, hogy jól fogok teljesíteni	6	5	4	3	2	1	0
K_18	Gyakran aggódom a közönség negatív reakciója miatt	0	1	2	3	4	5	6
K_19	Néha ok nélkül szorongok	0	1	2	3	4	5	6
K_20	Úgy emlékszem, már zenei tanulmányaim kezdetén is izgultam a fellépés miatt..	0	1	2	3	4	5	6
K_21	Aggódom, hogy egy rosszul sikerült fellépés tönkreteheti a zenei pályámat	0	1	2	3	4	5	6
K_22	Fellépés előtt/közben gyorsan kalapál a szívem.......................	0	1	2	3	4	5	6

		Egyáltalán nem értek egyet						Teljesen egyetértek
K_23	A szüleim majdnem mindig hallgattak rám	6	5	4	3	2	1	0
K_24	Kedvező fellépési lehetőségekről mondok le	0	1	2	3	4	5	6
K_25	Koncert után amiatt aggódom, hogy elég jól játszottame	0	1	2	3	4	5	6
K_26	A fellépés miatti aggodalmaskodás és az idegesség gátol abban, hogy koncentráljak	0	1	2	3	4	5	6
K_27	Gyerekként gyakran voltam szomorú	0	1	2	3	4	5	6
K_28	Koncert előtti felkészülésemet mindig félelem és a közelgő katasztrófa érzése kíséri	0	1	2	3	4	5	6
K_29	Valamelyik szülőm, vagy mindkét szülőm túlzottan aggodalmaskodó volt	0	1	2	3	4	5	6
K_30	Fellépés előtt vagy koncert közben az izmaim túlságosan megfeszülnek	0	1	2	3	4	5	6
K_31	Gyakran érzem azt, hogy semmit nem remélhetek	0	1	2	3	4	5	6
K_32	Koncert után az egészet visszajátszom magamban újra és újra	0	1	2	3	4	5	6
K_33	A szüleim bátorítottak arra, hogy próbáljak ki új dolgokat	6	5	4	3	2	1	0
K_34	Annyira izgulok koncert előtt, hogy nem tudok aludni	0	1	2	3	4	5	6
K_35	Amikor nem zenét adok elő, megbízható a memóriám	6	5	4	3	2	1	0
K_36	Koncert előtt vagy közben reszketek vagy remegek	0	1	2	3	4	5	6
K_37	Magabiztos vagyok, amikor kívülről kell valamit játszani	6	5	4	3	2	1	0
K_38	Aggaszt, ha mások megfigyelnek	0	1	2	3	4	5	6
K_39	Nyugtalanít az, hogy magam mit gondolok arról, hogyan fog sikerülni a fellépésem	0	1	2	3	4	5	6
K_40	Bár nagy izgalommal járnak, elkötelezett vagyok a fellépések irán	0	1	2	3	4	5	6

©Kenny, D.T. (2009). *Kenny Music Performance Anxiety Inventory-Revised* (K-MPAI-R)
