# Supplementary material for: The Kenny music performance anxiety inventory (K-MPAI): Scale construction, cross-cultural validation, theoretical underpinnings, and diagnostic and therapeutic utility
Source: Front Psychol. 2023 May 26;14:1143359. doi: 10.3389/fpsyg.2023.1143359 (PMC10262052; doi:10.3389/fpsyg.2023.1143359)
Supplement: Supplementary file 2 [file Data_Sheet_1.zip › K-MPAI_Italian translation.pdf]

Qui di seguito sono riportate alcune affermazioni su come ci si sente prima o durante un'esecuzione. Si prega di cerchiare il numero corrispondente secondo la propria opinone in relazione al condividere o meno le seguenti dichiarazioni.

		**Fortemente in disaccordo**						**Fortemente d'accordo**
K_1	Generalmente sento che ho il controllo della ma vita	6	5	4	3	2	1	0
K_2	Mi riesce facile fidarmi degli altri	6	5	4	3	2	1	0
K_3	A volte mi sento depresso senza sapere perché	0	1	2	3	4	5	6
K_4	Spesso mi riesce difficile trovare l'energia per fare le cose	0	1	2	3	4	5	6
K_5	La preoccupazione eccessiva é una caratterisica della mia famiglia	0	1	2	3	4	5	6
K_6	Ho spesso la sensazione che la vita non abbia molto da offrirmi	0	1	2	3	4	5	6
K_7	Anche se lavoro duramente nella preparazione di un'esecuzione, é probabile che commetta errori	0	1	2	3	4	5	6
K_8	Trovo difficoltà a dipendere dagli altri	0	1	2	3	4	5	6
K_9	I miei genitori erano per lo più sensibili alle mie esigenze	6	5	4	3	2	1	0
K_10	Prima o durante un'esecuzione, provo sentimenti simili al panico	0	1	2	3	4	5	6
K_11	Non so mai prima di un concerto se suonerò bene	0	1	2	3	4	5	6
K_12	Prima o durante un'esecuzione sento la bocca secca	0	1	2	3	4	5	6
K_13	Spesso sento di non valere molto come persona	0	1	2	3	4	5	6
K_14	Durante l'esecuzione mi ritrovo a pensare se riuscirò a portare a termine la performance	0	1	2	3	4	5	6
K_15	Il pensiero della valutazione che posso ricevere interferisce con la mia esecuzione	0	1	2	3	4	5	6
K_16	Prima o durante un'esecuzione, mi sento male, svenire, o sento torcere le budellla	0	1	2	3	4	5	6
K_17	Anche nelle situazioni di esecuzione pù stressanti, sono sicuro che suonerò bene	6	5	4	3	2	1	0
K_18	Sono spesso preoccupato per una reazione negativa del pubblico	0	1	2	3	4	5	6
K_19	A volte mi sento in ansia senza alcun motivo particolare	0	1	2	3	4	5	6
K_20	Fin dall'inizio dei miei studi musicali, ricordo di essere stato ansioso in relazione all'esecuzione	0	1	2	3	4	5	6

		Fortemente in disaccordo						Fortemente d’accordo
K_21	Mi preoccupo che una cattiva esecuzione possa rovinare la mia carriera	0	1	2	3	4	5	6
K_22	Prima o durante un’esecuzione, sento il cuore battere forte nel petto	0	1	2	3	4	5	6
K_23	I miei genitori mi hanno ascoltato quasi sempre	6	5	4	3	2	1	0
K_24	Rinuncio alle opportunitá di performance che valgono la pena a causa del’ansia	0	1	2	3	4	5	6
K_25	Dopo lo spettacolo, mi preoccupo se ho suonato abbastanza bene	0	1	2	3	4	5	6
K_26	La mia preoccupazione e il mio nervosismo per l’esecuzione interferiscono con la mia attenzione e concentrazione	0	1	2	3	4	5	6
K_27	Da bambino, spesso mi sentivo triste	0	1	2	3	4	5	6
K_28	Mi capita spesso di preparare un concerto con un senso di spavento e di disastro imminente	0	1	2	3	4	5	6
K_29	Uno o entrambi i miei genitori erano troppo ansiosi	0	1	2	3	4	5	6
K_30	Prima o durante una performance, ho aumentato la tensione muscolare	0	1	2	3	4	5	6
K_31	Spesso sento di non avere nulla per guardare al futuro	0	1	2	3	4	5	6
K_32	Dopo lo spettacolo, lo rivivo nella mia mente più e più volte	0	1	2	3	4	5	6
K_33	I miei genitori mi hanno incoraggiato a provare cose nuove	6	5	4	3	2	1	0
K_34	Mi preoccupo così tanto prima di una performance, da non riuscire a dormire	0	1	2	3	4	5	6
K_35	Quando suono senza partitura, la mia memoria è affidabile	6	5	4	3	2	1	0
K_36	Prima o durante la performance sperimento fremiti, tremoli o brividi	0	1	2	3	4	5	6
K_37	Quando suono a memoria mi sento sicuro	6	5	4	3	2	1	0
K_38	Mi preoccupo nel sapere di essere esaminato dagli altri	0	1	2	3	4	5	6
K_39	Mi preoccupo per il mio proprio giudizio su come suoneró	0	1	2	3	4	5	6
K_40	Continuo a perpetrare l’attivitá concertistica, anche se ciò é per me fonte di grande ansia	0	1	2	3	4	5	6

©Kenny, D.T. (2009). *Kenny Music Performance Anxiety Inventory-Revised* (K-MPAI-R)
Traduzione: Fiammetta e Rosanna Facchini
