# Supplementary material for: The Kenny music performance anxiety inventory (K-MPAI): Scale construction, cross-cultural validation, theoretical underpinnings, and diagnostic and therapeutic utility
Source: Front Psychol. 2023 May 26;14:1143359. doi: 10.3389/fpsyg.2023.1143359 (PMC10262052; doi:10.3389/fpsyg.2023.1143359)
Supplement: Supplementary file 2 [file Data_Sheet_1.zip › K-MPAI_Korean translation.pdf]

Korean Translation of K-MPAI (Kenny, 2009, 2011)

	전혀 동의 하지 않음					매우 동의함
1. 나는 일반적으로 내 삶을 통제할 수 있다고 느낀다.	6	5	4	3	2	1	0
2. 나는 다른 사람을 쉽게 믿는다.	6	5	4	3	2	1	0
3. 나는 때때로 이유없이 우울감을 느낀다.	0	1	2	3	4	5	6
4. 나는 종종 무언가를 하기 위해 에너지를 내기 어렵다.	0	1	2	3	4	5	6
5. 과도한 걱정은 유전적 특성이다	0	1	2	3	4	5	6
6. 나는 종종 삶을 통해 얻을 것이 없다고 느낀다.	0	1	2	3	4	5	6
7. 공연을 열심히 준비한다고 해도 실수가 나올 것 같다.	0	1	2	3	4	5	6
8. 나는 다른 사람들에게 의지하는 것이 어렵다.	0	1	2	3	4	5	6
9. 부모님은 대부분 나의 요구를 받아주셨다.	6	5	4	3	2	1	0
10. 공연 전이나 공연 중에 공황상태에 빠진다..	0	1	2	3	4	5	6
11. 나는 공연 전에 내가 잘 해낼거라고 생각하지 않는다.	0	1	2	3	4	5	6
12. 공연 전이나 공연 중에 입이 마른다.	0	1	2	3	4	5	6
13. 나는 종종 가치 없는 사람이라고 생각한다	0	1	2	3	4	5	6
14. 공연 중에 내가 그것을 이겨낼 수 있을지 생각하는 나 자신을 발견한다	0	1	2	3	4	5	6
15. 타인의 평가에 대한 걱정으로 공연에 영향을 받은 적이 있다	0	1	2	3	4	5	6
16. 공연 전이나 공연 중에 아프거나 기절할 것 같거나 속이 울렁거린다.	0	1	2	3	4	5	6
17. 스트레스가 심한 공연에서도 잘 해낼 수 있을 것이라고 자신한다.	6	5	4	3	2	1	0
18. 나는 종종 청중의 부정적 평가에 대해 걱정한다	0	1	2	3	4	5	6
19. 때때로 아무 이유없이 불안해진다.	0	1	2	3	4	5	6
20. 음악을 시작한 초기부터 공연에 대해 불안해했다.	0	1	2	3	4	5	6
21. 한번의 나쁜 공연이 경력에 오점을 남길 것 같다.	0	1	2	3	4	5	6
22. 연주 전이나 공연 중에 가슴이 두근거리는 것 같은 심장 박동수증가를 느낀다.	0	1	2	3	4	5	6
23. 나의 부모님은 내 말을 항상 경청했다.	6	5	4	3	2	1	0

Korean Translation of K-MPAI (Kenny, 2009, 2011)

24. 나는 불안 때문에 가치 있는 공연을 포기한다.	0	1	2	3	4	5	6
25. 공연이 끝난 후, 내가 충분히 잘했는지 걱정한다.	0	1	2	3	4	5	6
26. 공연에 대한 불안, 초조감이 집중력에 영향을 준다.	0	1	2	3	4	5	6
27. 어렸을 적에, 나는 종종 슬펐다	0	1	2	3	4	5	6
28. 나는 종종 두려움으로 공연을 준비한다.	0	1	2	3	4	5	6
29. 부모님 중, 한 분 또는 두 분 모두 걱정이 너무 많으셨다.	0	1	2	3	4	5	6
30. 공연 전이나 공연 중에 근육이 긴장이 증가한다.	0	1	2	3	4	5	6
31. 나는 종종 기대하는 바가 아무것도 없다고 느낀다.	0	1	2	3	4	5	6
32. 공연이 끝난 후, 계속 반복해서 공연을 마음속으로 다시 보기(replay)를 한다.	0	1	2	3	4	5	6
33. 나의 부모님은 새로운 것을 시도하기를 격려했다.	6	5	4	3	2	1	0
34. 공연 전에 걱정으로 잠을 이룰 수 없다	0	1	2	3	4	5	6
35. 음악없이 공연해도, 나의 기억력은 믿을 만하다.	6	5	4	3	2	1	0
36. 공연 전이나 공연 중에 신체적 떨림을 경험한다.	0	1	2	3	4	5	6
37. 나는 악보를 외워 연주하는데 자신이 있다.	6	5	4	3	2	1	0
38. 누군가 나를 세심히 평가하는 것이 부담스럽다.	0	1	2	3	4	5	
39. 내가 어떻게 공연할지에 대한 내 자신의 판단이 걱정된다.	0	1	2	3	4	5	6
40. 용기를 내어 연주를 해내었지만 높은 신체적 불안은 경험한다.	0	1	2	3	4	5	6

Copyright: Kenny, D. Y. (2009). Kenny Music Performance Anxiety Inventor-Revised (K- MPAI-R)

Translation: YD Institute.
