# Supplementary material for: The Kenny music performance anxiety inventory (K-MPAI): Scale construction, cross-cultural validation, theoretical underpinnings, and diagnostic and therapeutic utility
Source: Front Psychol. 2023 May 26;14:1143359. doi: 10.3389/fpsyg.2023.1143359 (PMC10262052; doi:10.3389/fpsyg.2023.1143359)
Supplement: Supplementary file 2 [file Data_Sheet_1.zip › K-MPAI_Latvian translation.pdf]

Kenijas mūzikas priekšnesuma trauksmes aptaujas pārskatītā versija latviešu valodā

Zemāk ir sniegti apgalvojumi par to, kā Jūs jūtaties kopumā un kā jūtaties **pirms uzstāšanās vai tās laikā**.
Lūdzu, apvelciet vienu ciparu, lai norādītu, cik ļoti piekrītat vai nepiekrītat katram apgalvojumam!

	Noteikti nepiekrītu						Noteikti piekrītu
**K_1** Kopumā man ir izjūta, ka es kontrolēju savu dzīvi	6	5	4	3	2	1	0
**K_2** Man ir viegli uzticēties citiem	6	5	4	3	2	1	0
**K_3** Reizēm es jūtos nomākts, nezinot kāpēc	0	1	2	3	4	5	6
**K_4** Man bieži vien ir grūti sakopot spēkus, lai kaut ko paveiktu/izdarītu	0	1	2	3	4	5	6
**K_5** Pārlieku liela raizēšanās ir raksturīga iezīme/īpašība manā ģimenē	0	1	2	3	4	5	6
**K_6** Man bieži šķiet, ka dzīvei nav daudz ko man piedāvāt	0	1	2	3	4	5	6
**K_7** Pat ja es smagi strādāju, gatavojoties priekšnesumam, pastāv iespēja, ka pieļaušu kļūdas	0	1	2	3	4	5	6
**K_8** Man ir grūti paļauties uz citiem	0	1	2	3	4	5	6
**K_9** Mani vecāki lielākoties reaģēja uz manām vajadzībām	6	5	4	3	2	1	0
**K_10** Pirms uzstāšanās vai tās laikā man rodas panikai līdzīgas izjūtas	0	1	2	3	4	5	6
**K_11** Pirms koncerta nekad nezinu to, vai uzstāšos labi	0	1	2	3	4	5	6
**K_12** Pirms uzstāšanās vai tās laikā man ir sausa mute	0	1	2	3	4	5	6
**K_13** Es bieži vien jūtos, ka man nav lielas personīgās vērtības (prasmes, talanti, zināšanas u.c.)	0	1	2	3	4	5	6
**K_14** Uzstāšanās laikā attopos, ka domāju par to, vai tikšu līdz priekšnesuma beigām	0	1	2	3	4	5	6
**K_15** Domas par to, kādu novērtējumu es varētu saņemt, traucē man uzstāties	0	1	2	3	4	5	6
**K_16** Pirms uzstāšanās vai tās laikā man ir slikta dūša vai ģībonis, vai kņudoņa vēderā	0	1	2	3	4	5	6
**K_17** Pat stresainas uzstāšanās laikā esmu pārliecināts, ka sniegšu labu priekšnesumu	6	5	4	3	2	1	0
**K_18** Es bieži vien raizējos par negatīvu reakciju no klausītājiem	0	1	2	3	4	5	6
**K_19** Dažreiz es izjūtu trauksmi bez īpaša iemesla	0	1	2	3	4	5	6
**K_20** Es atceros, ka jau agrīni savās mūzikas nodarbībās izjutu trauksmi par uzstāšanos	0	1	2	3	4	5	6

**K_21** Es uztraucos, ka viena neveiksmīga uzstāšanās varētu sabojāt manu karjeru	0	1	2	3	4	5	6
**K_22** Pirms uzstāšanās vai uzstāšanās laikā manas sirdsdarbības ātrums palielinās, un es jūtu, kā tā (sirds) sitas manās krūtīs.	0	1	2	3	4	5	6
**K_23** Mani vecāki gandrīz vienmēr manī ieklausījās	6	5	4	3	2	1	0
**K_24** Es atsakos no vērtīgām uzstāšanās iespējām	0	1	2	3	4	5	6
**K_25** Pēc uzstāšanās es uztraucos par to, vai nospēlēju/nodziedāju pietiekoši labi	0	1	2	3	4	5	6
**K_26** Manas bažas un uztraukums par manu uzstāšanos traucē man fokusēties un koncentrēties	0	1	2	3	4	5	6
**K_27** Kā bērns es bieži jutos bēdīgs	0	1	2	3	4	5	6
**K_28** Bieži vien es gatavojos koncertam, izjūtot šausmas un tuvojošos neveiksmi	0	1	2	3	4	5	6
**K_29** Viens vai abi mani vecāki bija pārlieku trauksmaini	0	1	2	3	4	5	6
**K_30** Pirms uzstāšanās vai tās laikā mani muskuļi kļūst saspringtāki	0	1	2	3	4	5	6
**K_31** Man bieži vien šķiet, ka man nav nekā, ko sagaidīt no dzīves	0	1	2	3	4	5	6
**K_32** Pēc uzstāšanās es to izspēlēju/izdziedu savās domās atkal un atkal	0	1	2	3	4	5	6
**K_33** Mani vecāki mani iedrošināja izmēģināt ko jaunu (piemēram, jaunus hobijus)	6	5	4	3	2	1	0
**K_34** Pirms uzstāšanās es uztraucos tik ļoti, ka nevaru gulēt	0	1	2	3	4	5	6
**K_35** Uzstājoties bez notīm, es varu paļauties uz savu atmiņu	6	5	4	3	2	1	0
**K_36** Pirms uzstāšanās vai tās laikā es piedzīvoju trīci, drebuļus vai trīsas	0	1	2	3	4	5	6
**K_37** Esmu pārliecināts, spēlējot/dziedot pēc atmiņas	6	5	4	3	2	1	0
**K_38** Man rada bažas tas, ka citi skrupulozi (sīki) pētīs manas kļūdas	0	1	2	3	4	5	6
**K_39** Man rada bažas manis paša vērtējums par to, cik labi es uzstāšos	0	1	2	3	4	5	6
**K_40** Es joprojām turpinu uzstāties, lai gan uzstāšanās mani biedē	0	1	2	3	4	5	6

Panti 1; 2; 9; 17; 23; 33; 35; 37 ir reversēti!

Apakšskalas	Iegūto baļļu skaits	%
1. Proksimālā somatiskā trauksme un satraukums par par uzstāšanos		
K_10 Pirms uzstāšanās vai tās laikā man rodas panikai līdzīgas izjūtas		
K_12 Pirms uzstāšanās vai tās laikā man ir sausa mute		
K_14 Uzstāšanās laikā attopos, ka domāju par to, vai tikšu līdz priekšnesuma beigām		
K_16 Pirms uzstāšanās vai tās laikā man ir slikta dūša vai ģībonis, vai kņudoņa vēderā		
K_22 Pirms uzstāšanās vai uzstāšanās laikā manas sirdsdarbības ātrums palielinās, un es jūtu, kā tā (sirds) sitas manās krūtīs.		
K_26 Manas bažas un uztraukums par manu uzstāšanos traucē man fokusēties un koncentrēties		
K_28 Bieži vien es gatavojos koncertam, izjūtot šausmas un tuvojošos neveiksmi		
K_30 Pirms uzstāšanās vai tās laikā mani muskuļi kļūst saspringtāki		
K_34 Pirms uzstāšanās es uztraucos tik ļoti, ka nevaru gulēt		
K_36 Pirms uzstāšanās vai tās laikā es piedzīvoju trīci, drebuļus vai trīsas		
K_40 Es joprojām turpinu uzstāties, lai gan uzstāšanās mani biedē		
**Kopsumma/66**		
2. Satraukums/bailes (negatīvas domas), kas ir vērstas uz sevis un citu pārbaudi		
K_7 Pat ja es smagi strādāju, gatavojoties priekšnesumam, pastāv iespēja, ka pieļaušu kļūdas		
K_15 Domas par to, kādu novērtējumu es varētu saņemt, traucē man uzstāties		
K_18 Es bieži vien raizējos par negatīvu reakciju no klausītājiem		
K_21 Es uztraucos, ka viena neveiksmīga uzstāšanās varētu sabojāt manu karjeru		
K_25 Pēc uzstāšanās es uztraucos par to, vai nospēlēju/nodziedāju pietiekoši labi		
K_32 Pēc uzstāšanās es to izspēlēju/izdziedu savās domās atkal un atkal		
K_38 Man rada bažas tas, ka citi skrupulozi (sīki) pētīs manas kļūdas		
K_39 Man rada bažas manis paša vērtējums par to, cik labi es uzstāšos		
**Kopsumma/48**		
3. Depresija/bezcerība (psiholoģiskā ievainojamība)		
K_1 Kopumā man ir izjūta, ka es kontrolēju savu dzīvi (-)*		
K_2 Man ir viegli uzticēties citiem (-)*		
K_3 Reizēm es jūtos nomākts, nezinot kāpēc		
K_4 Man bieži vien ir grūti sakopot spēkus, lai kaut ko paveiktu/izdarītu		

K_6 Man bieži šķiet, ka dzīvei nav daudz ko man piedāvāt		
K_8 Man ir grūti paļauties uz citiem		
K_13 Es bieži vien jūtos, ka man nav lielas personīgās vērtības (prasmes, talanti, zināšanas u.c.)		
K_31 Man bieži vien šķiet, ka man nav nekā, ko sagaidīt no dzīves		
**Kopsumma/48**		
4. Vecāku empātija		
K_9 Mani vecāki lielākoties reaģēja uz manām vajadzībām (-)*		
K_23 Mani vecāki gandrīz vienmēr manī ieklausījās (-)*		
K_27 Kā bērns es bieži jutos bēdīgs		
K_33 Mani vecāki mani iedrošināja izmēģināt ko jaunu (piemēram, jaunus hobijus) (-)*		
**Kopsumma/24**		
5. Atmiņa		
K_35 Uzstājoties bez notīm, es varu paļauties uz savu atmiņu (-)*		
K_37 Esmu pārliecināts, spēlējot/dziedot pēc atmiņas (-)*		
**Kopsumma/12**		
6. Trauksmes pārnešana no paaudzes paaudzē		
K_5 Pārlieku liela raizēšanās ir raksturīga iezīme/īpašība manā ģimenē		
K_19 Dažreiz es izjūtu trauksmi bez īpaša iemesla		
K_29 Viens vai abi mani vecāki bija pārlieku trauksmaini		
**Kopsumma/18**		
7. Trauksmainas bažas		
K_11 Pirms koncerta nekad nezinu to, vai uzstāšos labi		
K_17 Pat stresainas uzstāšanās laikā esmu pārliecināts, ka sniegšu labu priekšnesumu (-)*		
K_24 Es atsakos no vērtīgām uzstāšanās iespējām		
**Kopsumma/18**		
8. Bioloģsikā ievainojamība		
K_20 Es atceros, ka jau agrīni savās mūzikas nodarbībās izjutu trauksmi par uzstāšanos		
**Kopsumma/6**		
**Kopējais iespējamais baļļu skaits/240**		
