# Supplementary material for: The Kenny music performance anxiety inventory (K-MPAI): Scale construction, cross-cultural validation, theoretical underpinnings, and diagnostic and therapeutic utility
Source: Front Psychol. 2023 May 26;14:1143359. doi: 10.3389/fpsyg.2023.1143359 (PMC10262052; doi:10.3389/fpsyg.2023.1143359)
Supplement: Supplementary file 2 [file Data_Sheet_1.zip › K-MPAI_Lithuanian translation.pdf]

Kenny Muzikos atlikimo nerimo klausimynas (KMPAI, 2009)

Prie kiekvieno teiginio apibraukite skaičių, geriausiai atspindintį Jūsų nuomonę.

	Visiškai nesutinku						*Visiškai sutinku*
1. Paprastai jaučiu, kad kontroliuoju savo gyvenimą.	6	5	4	3	2	1	0
2. Man lengva pasitikėti kitais.	6	5	4	3	2	1	0
3. Kartais jaučiuosi prislėgtas (-a), net nežinodamas (-a) kodėl.	0	1	2	3	4	5	6
4. Kai reikia ką nors atlikti, man dažnai būna sunku sukaupti jėgas.	0	1	2	3	4	5	6
5. Perdėtas nerimas yra būdingas mano šeimos bruožas.	0	1	2	3	4	5	6
6. Dažnai jaučiu, kad gyvenimas nelabai ką gali man pasiūlyti.	0	1	2	3	4	5	6
7. Net jei daug dirbu ruošdamasis pasirodymui, vis tiek nepavyksta išvengti klaidų.	0	1	2	3	4	5	6
8. Man sunku būti priklausomam (-ai) nuo kitų.	0	1	2	3	4	5	6
9. Mano tėvai dažniausiai būdavo jautrūs mano poreikiams.	6	5	4	3	2	1	0
10. Prieš pasirodymą ar jo metu išgyvenu pojūčius, panašius į paniką.	0	1	2	3	4	5	6
11. Prieš koncertą niekada nežinau, ar pasirodysiu gerai.	0	1	2	3	4	5	6
12. Prieš pasirodymą ar jo metu man džiūsta burna.	0	1	2	3	4	5	6
13. Dažnai jaučiu, kad esu nelabai ko vertas (-a).	0	1	2	3	4	5	6
14. Pasirodymo metu dažnai pagalvoju ar man pavyks jį sėkmingai užbaigti.	0	1	2	3	4	5	6
15. Kai galvoju apie galimą įvertinimą, tai trukdo mano pasirodymui.	0	1	2	3	4	5	6
16. Prieš pasirodymą ar jo metu man būna silpna, alpstu arba susuka vidurius.	0	1	2	3	4	5	6
17. Net ir daugiausiai įtampos keliančiuose pasirodymuose būnu įsitikinęs (-usi), kad pasirodysiu gerai.	6	5	4	3	2	1	0
18. Aš dažnai nerimauju dėl neigiamos auditorijos reakcijos.	0	1	2	3	4	5	6
19. Kartais be jokios konkrečios priežasties mane apima nerimas.	0	1	2	3	4	5	6

20. Atsimenu, kad jau muzikos mokslų pradžioje nerimaudavau dėl savo pasirodymų.	0	1	2	3	4	5	6
21. Man baisu, kad vienas prastas pasirodymas gali sužlugdyti visą mano karjerą.	0	1	2	3	4	5	6
22. Prieš pasirodymą ar jo metu man smarkiai daužosi krūtinėje širdis, tarsi padažnėja pulsas.	0	1	2	3	4	5	6
23. Mano tėvai visada mane išklausydavo.	6	5	4	3	2	1	0
24. Dėl nerimo dažnai atsisakau vertingų progų pasirodyti.	0	1	2	3	4	5	6
25. Po pasirodymo dažnai jaudinuosi, ar pakankamai gerai grojau.	0	1	2	3	4	5	6
26. Nerimas ir nervingumas dėl pasirodymo man trukdo susikaupti ir susikoncentruoti.	0	1	2	3	4	5	6
27. Vaikystėje man dažnai būdavo liūdna.	0	1	2	3	4	5	6
28. Dažnai ruošiuosi koncertui su baime ir gresiančios nesėkmės nuojauta.	0	1	2	3	4	5	6
29. Vienas ar abu mano tėvai būdavo linkę pernelyg nerimauti.	0	1	2	3	4	5	6
30. Prieš pasirodymą ar jo metu man smarkiai įsitempia raumenys.	0	1	2	3	4	5	6
31. Dažnai jaučiuosi taip, tarsi ateity man nebėra ko laukti.	0	1	2	3	4	5	6
32. Po pasirodymo daug kartų kartoju jį mintyse.	0	1	2	3	4	5	6
33. Mano tėvai skatindavo mane išmėginti naujus dalykus.	6	5	4	3	2	1	0
34. Aš taip jaudinuosi prieš pasirodymą, kad net negaliu užmigti.	0	1	2	3	4	5	6
35. Atlikdamas kūrinį be muzikos (mintyse), galiu pasikliauti savo atmintimi.	0	1	2	3	4	5	6
36. Prieš pasirodymą ar jo metu aš visas (-a) drebu, virpu.	0	1	2	3	4	5	6
37. Pasitikiu savim kai tenka groti atmintinai.	6	5	4	3	2	1	0
38. Man neramu dėl to, kad mane atidžiai stebės kiti.	0	1	2	3	4	5	6
39. Man neramu dėl to, kaip aš pats įvertinsiu savo pasirodymą.	0	1	2	3	4	5	6
40. Aš neatsisakau pasirodymų, net jei tai man sukelia didelį nerimą.	0	1	2	3	4	5	6

Translated by Vilma Paliaukiene
