# Supplementary material for: The Kenny music performance anxiety inventory (K-MPAI): Scale construction, cross-cultural validation, theoretical underpinnings, and diagnostic and therapeutic utility
Source: Front Psychol. 2023 May 26;14:1143359. doi: 10.3389/fpsyg.2023.1143359 (PMC10262052; doi:10.3389/fpsyg.2023.1143359)
Supplement: Supplementary file 2 [file Data_Sheet_1.zip › K-MPAI_Persian translation.pdf]

پرسشنامه اضطراب اجرای موسیقی کِنی (K-MPAIR)

عبارات زیر احساس عمومي شما و همچنین احساستان قبل از اجرا و در حین اجراي موسیقي را مورد پرسش قرار مي دهد. لطفاَ میزان موافقت و عدم موافقت خود با هر عبارت را با انتخاب یك عدد نشان دهید.

معمولا احساس می کنم که زندگی ام تحت کنترلم است............

من به راحتی به دیگران اعتماد میکنم..........................

گاهی اوقات بدون اینکه بدانم چرا، احساس افسردگی می کنم..

اغلب برایم سخت است تا انرژی لازم را برای انجام کارهایم فراهم کنم..

نگرانی بیش از حد، یکی از ویژگیهای خانواده ی من است..........

من اغلب احساس میکنم که زندگی چیز بیشتری ندارد به من عرضه کند..

حتی اگر برای آمادگی در یک اجرا به شدت تلاش کنم ، باز هم احتمال میدهم ک
شوم..

برای من سخت است که به دیگران متکی باشم.......................

والدینم بیش از همه به نیازهایم پاسخگو بودند..................

قبل ، یا در طول اجرا دچار احساساتی شبیه به وحشت می شوم..

هرگز قبل از یک کنسرت نمیدانم که آیا اجرای خوبی خواهم داشت یا نه..

قبل ، یا در طول اجرا دهانم خشک می شود......................

من اغلب احساس میکنم که به عنوان یک فرد ارزش ندارم..........

در طول یک اجرا متوجه میشوم در حال فکر کردن به این هستم که آیا آن را ب
نه..

فکر کردن در مورد ارزشیابی ممکن است در اجرای من تداخل ایجاد کند..

قبل ،یا در طول اجرا احساس بیماری و ضعف و همچنین احساس بیقراری و آشوب
میکنم..

حتی در پر استرس ترین شرایط اجرا، مطمئن هستم که اجرای خوبی خواهم داشت ....

من اغلب نگران واکنش منفی از طرف مخاطبین هستم..............

گاهی اوقات بدون هیچ دلیل خاصی احساس نگرانی میکنم..........

بهیاد دارم که از ابتدای یادگیری موسیقی، نگران اجرا کردن بودم.

نگران این هستم که یک اجرای بد جایگاه شغلی من را خراب خواهد کرد...
قبل، یا در طول اجرا ضربان قلبم افزایش پیدا میکند و تپش را در سینه ام میکنم...
والدینم تقریبا ، همیشه به من گوش داده اند.........

من از فرصت های اجرای ارزشمندی منصرف میشوم...
بعد از اجرا، نگران این هستم که آیا به اندازه کافی خوب نواختم یا نه...
نگرانی و عصبی بودنم در باره اجرا کردن ،در تمرکز من تداخل ایجاد می کند...
در کودکی اغلب غمگین بودم.........................

اغلب با احساس ترس ورخدادن یک فاجعه برای کنسرت آماده میشوم...
یکی از والدینم یا هر دو آنها بیش از اندازه مضطرب بودند.........

قبل یا در طول اجرا دچار تنش عضلانی میشوم..................

من اغلب احساس می‌کنم هیچ چیزی ندارم که مشتاقانه منتظرش باشم...
بعد از اجرا، بارها وبارها آنرا در ذهنم تکرار می‌کنم...............

والدینم مرا به امتحان کردن چیز های جدید تشویق کردند..........

من قبل از اجرا خیلی دلواپسم ، نمی‌توانم بخوابم....................

زمانی که بدون موسیقی اجرا میکنم، حافظه ام قابل اعتماد است.

قبل یا در طول اجرا ، دچار لرزش یا رعشه می‌شوم.................

من در اجرا کردنِ از حفظ، اعتماد به نفس دارم.....................

من از اینکه توسط دیگران مورد بررسی دقیق قرار بگیرم نگرانم...

من از قضاوت خودم راجع به اینکه چگونه اجرا خواهم کرد نگرانم...
من به اجرا کردن متعهد خواهم ماند حتی اگر دچار اضطراب فراوان شوم...
