# Supplementary material for: The Kenny music performance anxiety inventory (K-MPAI): Scale construction, cross-cultural validation, theoretical underpinnings, and diagnostic and therapeutic utility
Source: Front Psychol. 2023 May 26;14:1143359. doi: 10.3389/fpsyg.2023.1143359 (PMC10262052; doi:10.3389/fpsyg.2023.1143359)
Supplement: Supplementary file 2 [file Data_Sheet_1.zip › K-MPAI_Polish translation.pdf]

Kenny Music Performance Anxiety Inventory-Revised (K-MPAI-R)
wersja polska

Poniżej znajdują się stwierdzenia na temat tego, jak się zwykle czujesz oraz jak się czujesz **przed i w trakcie swojego występu**. Przy każdym stwierdzeniu zakreśl jedną cyfrę, aby wskazać, w jakim stopniu się z nim zgadzasz.

		Zdecydowanie się nie zgadzam					Zdecydowanie się zgadzam	
1	Zwykle czuję, że mam kontrolę nad swoim życiem.	6	5	4	3	2	1	0
2	Jest mi łatwo zaufać innym.	6	5	4	3	2	1	0
3	Czasami czuję się przygnębiony/przygnębiona, nie wiedząc dlaczego.	0	1	2	3	4	5	6
4	Często jest mi trudno wykrzesać energię, żeby coś zrobić.	0	1	2	3	4	5	6
5	Nadmierne zamartwianie się to cecha charakterystyczna mojej rodziny.	0	1	2	3	4	5	6
6	Często czuję, że życie ma mi niewiele do zaoferowania.	0	1	2	3	4	5	6
7	Nawet jeżeli ciężko pracuję przygotowując się do występu, i tak najprawdopodobniej popełnię błąd.	0	1	2	3	4	5	6
8	Jest mi trudno polegać na kimś innym.	0	1	2	3	4	5	6
9	Moi rodzice przeważnie byli wrażliwi na moje potrzeby.	6	5	4	3	2	1	0
10	Przed lub w trakcie występu mam odczucia podobne do paniki.	0	1	2	3	4	5	6
11	Przed koncertem nigdy nie wiem, czy dobrze wypadnę.	0	1	2	3	4	5	6
12	Przed lub w trakcie występu odczuwam suchość w ustach.	0	1	2	3	4	5	6
13	Często czuję, że jestem niewiele wart/warta jako człowiek.	0	1	2	3	4	5	6
14	Podczas występu zastanawiam się, czy w ogóle przez niego przebrnę.	0	1	2	3	4	5	6
15	Świadomość tego, że ktoś mnie ocenia, zakłóca mój występ.	0	1	2	3	4	5	6
16	Przed lub w trakcie występu jest mi niedobrze, czuję się słabo lub mam sensacje żołądkowe.	0	1	2	3	4	5	6
17	Nawet podczas najbardziej stresującego występu jestem pewny/pewna, że wypadnę dobrze.	6	5	4	3	2	1	0
18	Często obawiam się negatywnej reakcji ze strony publiczności.	0	1	2	3	4	5	6

19	Czasami odczuwam lęk bez żadnego konkretnego powodu.	0	1	2	3	4	5	6
20	Pamiętam, że od początku mojej edukacji muzycznej towarzyszył mi lęk związany z występowaniem.	0	1	2	3	4	5	6
21	Martwię się, że jeden zły występ może zrujnować moją karierę.	0	1	2	3	4	5	6
22	Przed lub w trakcie występu odczuwam przyspieszone bicie serca, jakby łomotanie w klatce piersiowej.	0	1	2	3	4	5	6
23	Moi rodzice prawie zawsze wysłuchiwali mnie.	6	5	4	3	2	1	0
24	Wycofuję się, gdy mam okazję wziąć udział w jakimś ważnym występie.	0	1	2	3	4	5	6
25	Po występie martwię się czy zagrałem/zagrałam wystarczająco dobrze.	0	1	2	3	4	5	6
26	Moje obawy i zdenerwowanie związane z występem przeszkadzają mi w skupieniu i koncentracji.	0	1	2	3	4	5	6
27	Jako dziecko często byłem/byłam smutny/smutna.	0	1	2	3	4	5	6
28	Często przygotowuję się do koncertu z przerażeniem i poczuciem zbliżającej się katastrofy.	0	1	2	3	4	5	6
29	Moi rodzice, lub jedno z nich, są/byli nadmiernie lękliwi.	0	1	2	3	4	5	6
30	Przed lub w trakcie występu mam spięte mięśnie.	0	1	2	3	4	5	6
31	Często czuję, że nic dobrego mnie nie czeka.	0	1	2	3	4	5	6
32	Gdy skończy się występ, wielokrotnie odtwarzam go w myślach.	0	1	2	3	4	5	6
33	Moi rodzice zachęcali mnie do próbowania nowych rzeczy.	6	5	4	3	2	1	0
34	Przed występem martwię się tak bardzo, że nie mogę spać.	0	1	2	3	4	5	6
35	Gdy na koncercie gram bez nut, moja pamięć mnie nie zawodzi.	6	5	4	3	2	1	0
36	Przed lub w trakcie występu drżę, trzęsę się lub mam dreszcze.	0	1	2	3	4	5	6
37	Jestem pewny/pewna siebie, kiedy gram z pamięci.	6	5	4	3	2	1	0
38	Martwię się, gdy ktoś mnie wnikliwie ocenia.	0	1	2	3	4	5	6
39	Martwię się tym, jak sam/sama ocenię swój występ.	0	1	2	3	4	5	6
40	Nie przestaję występować mimo, że powoduje to u mnie duży lęk.	0	1	2	3	4	5	6
