# Supplementary material for: The Kenny music performance anxiety inventory (K-MPAI): Scale construction, cross-cultural validation, theoretical underpinnings, and diagnostic and therapeutic utility
Source: Front Psychol. 2023 May 26;14:1143359. doi: 10.3389/fpsyg.2023.1143359 (PMC10262052; doi:10.3389/fpsyg.2023.1143359)
Supplement: Supplementary file 2 [file Data_Sheet_1.zip › K-MPAI_Portuguese translation.pdf]

A seguir, estão relacionadas questões que, em linhas gerais, expressam como você se sente durante, ou antes, de uma apresentação. Por favor, marque o número queindique o quanto você concorda ou discorda das afirmações.

	Discordo plenamente						Concordo plenamente
1. Geralmente sinto que tenho minha vida sob controle.	6	5	4	3	2	1	0
2. Confio facilmente em outras pessoas.	6	5	4	3	2	1	0
3. Às vezes me sinto deprimido sem saber por quê.	0	1	2	3	4	5	6
4. Acho difícil reunir forças para realizar tarefas.	0	1	2	3	4	5	6
5. Preocupação excessiva é característica comum em minha família.	0	1	2	3	4	5	6
6. Frequentemente, sinto que a vida não tem muito a me oferecer.	0	1	2	3	4	5	6
7. Quanto mais preparo uma peça para uma apresentação, mais cometo erros graves.	0	1	2	3	4	5	6
8. Sinto dificuldades em depender de outras pessoas.	0	1	2	3	4	5	6
9. Meus pais frequentemente eram compreensíveis e atenciosos com relação às minhas demandas.	6	5	4	3	2	1	0
10. Tenho sensações de pânico antes ou durante as apresentações.	0	1	2	3	4	5	6
11. Nunca posso prever se minha apresentação será um sucesso.	0	1	2	3	4	5	6
12. Antes ou durante uma apresentação, sinto garganta e boca secarem.	0	1	2	3	4	5	6
13. Frequentemente, sinto que não tenho tanto valor enquanto indivíduo.	0	1	2	3	4	5	6
14. Durante uma apresentação, começo a pensar se serei capaz de chegar até o fim da peça.	0	1	2	3	4	5	6
15. Pensar sobre como eu possa ser avaliado interfere em minha apresentação.	0	1	2	3	4	5	6
16. Antes ou durante uma apresentação, sinto mal-estar estomacal ou vertigens.	0	1	2	3	4	5	6
17. Mesmo nas apresentações mais estressantes, tenho confiança de que me sairei bem.	6	5	4	3	2	1	0
18. Frequentemente me preocupo com uma reação negativa da plateia.	0	1	2	3	4	5	6
19. Às vezes me sinto ansioso sem motivo aparente.	0	1	2	3	4	5	6
20. Desde o início de minha carreira musical, lembro-me de estar sempre nervoso em apresentações.	0	1	2	3	4	5	6
21. Preocupo-me que uma má apresentação possa arruinar minha carreira.	0	1	2	3	4	5	6
22. Antes ou durante uma apresentação, sinto aumento da frequência cardíaca como um pulsar forte no peito.	0	1	2	3	4	5	6
23. Quase sempre fui ouvido por meus pais.	6	5	4	3	2	1	0
24. Eu desisto de boas oportunidades de apresentação em virtude da ansiedade.	0	1	2	3	4	5	6
25. Após uma apresentação, eu sempre me pergunto se minha *performance* foi boa o suficiente.	0	1	2	3	4	5	6
26. Minha preocupação e nervosismo sobre a interpretação interferem na minha concentração.	0	1	2	3	4	5	6
27. Quando criança, frequentemente me sentia triste.	0	1	2	3	4	5	6
28. Frequentemente me preparo para um concerto com um sentimento de desastre iminente, ou mau presságio.	0	1	2	3	4	5	6
29. Um de meus pais ou ambos eram muito ansiosos.	0	1	2	3	4	5	6
30. Sinto aumento na tensão muscular antes ou durante uma apresentação.	0	1	2	3	4	5	6
31. Frequentemente, sinto que o futuro não me trará alegrias.	0	1	2	3	4	5	6
32. Após terminar a apresentação, continuo repetindo-a em minha mente.	0	1	2	3	4	5	6
33. Meus pais me estimularam a tentar coisas novas.	6	5	4	3	2	1	0
34. Preocupo-me tanto antes de uma apresentação que não consigo dormir.	0	1	2	3	4	5	6
35. Quando toco sem a partitura, considero minha memória confiável.	6	5	4	3	2	1	0
36. Antes ou durante uma apresentação, sinto tremores no corpo.	0	1	2	3	4	5	6
37. Sinto-me confiante tocando de memória.	6	5	4	3	2	1	0
38. Preocupa-me ser "examinado" por outras pessoas.	0	1	2	3	4	5	6
39. Eu me preocupo com o meu próprio julgamento acerca de como será a minha *performance*	0	1	2	3	4	5	6
40. Permaneço engajado com as apresentações, mesmo me causando grande ansiedade.	0	1	2	3	4	5	6

©Sérgio Rocha (2011) – Versão em português da K-MPAI de Kenny DT (2009).
