# Supplementary material for: The Kenny music performance anxiety inventory (K-MPAI): Scale construction, cross-cultural validation, theoretical underpinnings, and diagnostic and therapeutic utility
Source: Front Psychol. 2023 May 26;14:1143359. doi: 10.3389/fpsyg.2023.1143359 (PMC10262052; doi:10.3389/fpsyg.2023.1143359)
Supplement: Supplementary file 2 [file Data_Sheet_1.zip › K-MPAI_Romanian translation.pdf]

K-MPAI

Chestionarul de mai jos investighează mai multe aspecte legate de emoţiile pe care le ai atunci când urmează să cânţi în faţa unui public. Te rugăm să marchezi răspunsul care descrie cel mai bine felul în care gândeşti în legătură cu aceste aspecte. Nu există răspunsuri greşite.

Răspunsurile pot varia între 0 - deloc (această afirmaţie nu descrie felul în care gândesc sau mă simt) şi 6- foarte mult (această afirmaţie descrie foarte bine felul în care gândesc sau mă simt).

Item	Deloc						Foarte mult
1) În general, simt că am control asupra vieții mele.	6	5	4	3	2	1	0
2) Mi-e ușor să am încredere în alții.	6	5	4	3	2	1	0
3) Uneori mă simt deprimat(ă) fără să știu de ce.	0	1	2	3	4	5	6
4) Adesea mi se pare dificil să găsesc energia de a face diverse lucruri.	0	1	2	3	4	5	6
5) Îngrijorarea excesivă este o caracteristică în familia mea.	0	1	2	3	4	5	6
6) Adesea simt că viața nu are multe să-mi ofere.	0	1	2	3	4	5	6
7) Chiar dacă mă pregătesc temeinic pentru o reprezentație scenică, pot face greșeli.	0	1	2	3	4	5	6
8) Mi se pare dificil să depind de alții.	0	1	2	3	4	5	6
9) Părinții mei au fost în cea mai mare partea a timpului sensibili la nevoile mele.	6	5	4	3	2	1	0
10) Înainte de sau în timpul unui concert, am stări asemănătoare cu panica.	0	1	2	3	4	5	6
11) Nu știu niciodată, înaintea unui concert, dacă voi cânta bine.	0	1	2	3	4	5	6
12) Înainte de sau în timpul unui concert, simt că am gura uscată.	0	1	2	3	4	5	6
13) Simt adesea că nu sunt o persoană valoroasă.	0	1	2	3	4	5	6
14) În timpul unei reprezentații scenice, mă îngrijorez dacă voi reuși să o duc la final.	0	1	2	3	4	5	6
15) Atunci când mă gândesc negativ la evaluare, acest lucru poate interfera cu performanța mea.	0	1	2	3	4	5	6
16) Înainte de sau în timpul unui concert, mă simt rău sau simt o stare de leșin sau de agitație în stomac.	0	1	2	3	4	5	6
17) Chiar și în cele mai stresante situații de reprezentație scenică, sunt încrezător/încrezătoare că mă voi descurca bine.	6	5	4	3	2	1	0
18) Sunt adesea preocupat/ă de o reacție negativă din partea publicului.	0	1	2	3	4	5	6
19) Uneori mă simt anxios/anxioasă fără un motiv anume.	0	1	2	3	4	5	6
20) Încă de la începutul studiilor mele muzicale, țin minte că eram anxios/oasă în ceea ce privește cântatul.	0	1	2	3	4	5	6

Item	Deloc						Foarte mult
21) Mă îngrijorez că o reprezentație scenică slabă îmi poate distruge cariera.	0	1	2	3	4	5	6
22) Înainte de sau în timpul unui concert, simt cum îmi crește ritmul cardiac și inima îmi bate puternic în piept.	0	1	2	3	4	5	6
23) Părinții mei m-au ascultat întotdeauna.	6	5	4	3	2	1	0
24) Din cauza anxietății, renunț la oportunități importante de a performa.	0	1	2	3	4	5	6
25) După o reprezentație scenică, mă îngrijorez dacă am cântat suficient de bine.	0	1	2	3	4	5	6
26) Îngrijorările și anxietatea legate de reprezentația mea scenică îmi afectează în mod negativ atenția și concentrarea.	0	1	2	3	4	5	6
27) Copil fiind, adesea mă simțeam trist.	0	1	2	3	4	5	6
28) Adesea mă pregătesc pentru o reprezentație scenică cu o stare de teamă și dezastru iminent.	0	1	2	3	4	5	6
29) Unul sau ambii părinți au fost exagerat de anxioși.	0	1	2	3	4	5	6
30) Înainte de sau în timpul unui concert, am o stare crescută de tensiune musculară.	0	1	2	3	4	5	6
31) Adesea simt că nu am nimic la ce să mă aștept pe viitor.	0	1	2	3	4	5	6
32) După un concert, îl repet în mintea mea la nesfârșit.	0	1	2	3	4	5	6
33) Părinții mei m-au încurajat să încerc lucruri noi.	6	5	4	3	2	1	0
34) Înaintea unei reprezentații scenice, mă îngrijorez atât de mult, încât un pot dormi.	0	1	2	3	4	5	6
35) Atunci când cânt fără partitură, mă pot baza pe memoria mea.	6	5	4	3	2	1	0
36) Înainte de sau în timpul unui concert, simt fiori, simt că tremur.	0	1	2	3	4	5	6
37) Am încredere că pot cânta pe dinafară.	6	5	4	3	2	1	0
38) Sunt preocupat(ă) de faptul că ceilalți m-ar evalua amănunțit.	0	1	2	3	4	5	6
39) Sunt preocupat(ă) de propria judecată privind cum voi performa.	0	1	2	3	4	5	6
40) Sunt hotărât(ă) să performez, chiar dacă acest lucru mă îngrozește.	0	1	2	3	4	5	6

Translated by Andrada Jucan
