# Supplementary material for: The Kenny music performance anxiety inventory (K-MPAI): Scale construction, cross-cultural validation, theoretical underpinnings, and diagnostic and therapeutic utility
Source: Front Psychol. 2023 May 26;14:1143359. doi: 10.3389/fpsyg.2023.1143359 (PMC10262052; doi:10.3389/fpsyg.2023.1143359)
Supplement: Supplementary file 2 [file Data_Sheet_1.zip › K-MPAI_Slovenian translation.pdf]

Slovenian adaptation of K-MPAI-R (Kenny Music Performance Anxiety Inventory-Revised, 2009)
Adapted by: Kaja Pojbic, Ana Gregorec, Urban Stiberc and Zala Brecko

		Močno se ne strinjam						Močno se strinjam
1.	V splošnem čutim, da imam življenje pod nadzorom……………......	6	5	4	3	2	1	0
2.	Z lahkoto zaupam drugim. ……………......………………..................	6	5	4	3	2	1	0
3.	Včasih se počutim depresivno, ne da bi vedel/-a zakaj. …………….	0	1	2	3	4	5	6
4.	Pogosto le s težavo najdem energijo, da bi kaj počel/-a…………….	0	1	2	3	4	5	6
5.	Za mojo družino je značilna pretirana zaskrbljenost………………..	0	1	2	3	4	5	6
6.	Pogosto imam občutek, da mi življenje ne ponuja veliko…………..	0	1	2	3	4	5	6
7.	Čeprav se s trdim delom pripravljam na nastop, bom verjetno storil/-a napake. ………………………………………………………..	0	1	2	3	4	5	6
8.	Težko se zanesem na druge……………….……………......………………..	0	1	2	3	4	5	6
9.	Starši so se večinoma odzivali na moje potrebe. ……………...........	6	5	4	3	2	1	0
10.	Pred ali med nastopom doživljam paniki podobne občutke. ………..	0	1	2	3	4	5	6
11.	Pred nastopom nikoli ne vem, ali se bom dobro odrezal/-a. ………..	0	1	2	3	4	5	6
12.	Pred ali med nastopom imam suha usta. …………….........................	0	1	2	3	4	5	6
13.	Pogosto čutim, da kot oseba nisem veliko vreden/-a. …………….....	0	1	2	3	4	5	6
14.	Med nastopom se sprašujem, ali se bom prebil/-a do konca………..	0	1	2	3	4	5	6
15.	Ko pomislim, da me poslušalci ocenjujejo, to slabo vpliva na moj nastop. ……………......……………......……………......………..	0	1	2	3	4	5	6
16.	Pred ali med nastopom mi je slabo, omedlim ali me zvija v trebuhu..	0	1	2	3	4	5	6
17.	Tudi pri najbolj stresnih nastopih sem prepričan/-a, da bom nastop dobro izvedel/-a. ……………......……………......…………………	6	5	4	3	2	1	0
18.	Pogosto sem zaskrbljen/-a glede negativnega odziva občinstva……	0	1	2	3	4	5	6
19.	Včasih se počutim tesnobno brez pravega razloga………………..	0	1	2	3	4	5	6
20.	Spomnim se, da se nastopanja bojim že od samih začetkov mojega glasbenega izobraževanja. ……………......……………….................	0	1	2	3	4	5	6
21.	Skrbi me, da bi mi slab nastop uničil mojo kariero. ……………......	0	1	2	3	4	5	6
22.	Pred ali med nastopom mi razbija srce. ……………........................	0	1	2	3	4	5	6
23.	Starši so me skoraj vedno poslušali. ……………......……………....	6	5	4	3	2	1	0
24.	Odpovem se dobrim priložnostim za nastopanje……………………	0	1	2	3	4	5	6
25.	Po nastopu me skrbi, ali sem igral/-a dovolj dobro. ………………..	0	1	2	3	4	5	6
26.	Moje skrbi in živčnost glede nastopa vplivajo na mojo osredotočenost in koncentracijo. ……………................................	0	1	2	3	4	5	6
27.	Kot otrok sem bil/-a pogosto žalosten/-a. ………………..................	0	1	2	3	4	5	6
28.	Na koncert se pogosto pripravljam z občutkom groze in bližajoče se katastrofe. ……………......……………......……………......……….	0	1	2	3	4	5	6
29.	Eden ali oba starša sta bila pretirano tesnobna. …………..............	0	1	2	3	4	5	6
30.	Pred ali med nastopom so moje mišice bolj napete. ……………......	0	1	2	3	4	5	6
31.	Pogosto imam občutek, da se nimam ničesar veseliti. ………………	0	1	2	3	4	5	6
32.	Ko je nastop že za mano, si ga v glavi večkrat ponavljam. …………	0	1	2	3	4	5	6
33.	Starši so me spodbujali k poskušanju novih stvari. ……………......	6	5	4	3	2	1	0
34.	Pred nastopom sem tako zaskrbljen/-a, da ne morem spati. ………..	0	1	2	3	4	5	6
35.	Kadar nastopam v drugih (neglasbenih) situacijah, je moj spomin zanesljiv. ……………......……………......……………......………..	6	5	4	3	2	1	0
36.	Pred ali med nastopom drhtim ali se tresem. ……………................	0	1	2	3	4	5	6
37.	Zaupam vase, ko igram po spominu. ……………......……………....	6	5	4	3	2	1	0
38.	Skrbi me, da bom pod drobnogledom poslušalcev. ……………......	0	1	2	3	4	5	6
39.	Skrbi me, kako uspešen se mi bo zdel moj nastop. ……………......	0	1	2	3	4	5	6
40.	Še vedno sem predan/a nastopanju, čeprav me navdaja s tesnobo…..	0	1	2	3	4	5	6
