# Supplementary material for: The Kenny music performance anxiety inventory (K-MPAI): Scale construction, cross-cultural validation, theoretical underpinnings, and diagnostic and therapeutic utility
Source: Front Psychol. 2023 May 26;14:1143359. doi: 10.3389/fpsyg.2023.1143359 (PMC10262052; doi:10.3389/fpsyg.2023.1143359)
Supplement: Supplementary file 2 [file Data_Sheet_1.zip › K-MPAI_Spanish translation.pdf]

A continuación se te presentan algunas afirmaciones acerca de cómo te sientes generalmente y cómo te sientes **durante o antes de una presentación**. Por favor, encierra en un círculo el número que indique cuán de acuerdo o en desacuerdo estás con cada afirmación.

		**Totalmente en desacuerdo**						**Totalmente de acuerdo**
K_1	En general, me siento en control de mi vida	6	5	4	3	2	1	0
K_2	Me es fácil confiar en los demás	6	5	4	3	2	1	0
K_3	Algunas veces me siento deprimido(a) sin saber por qué	0	1	2	3	4	5	6
K_4	A menudo me es difícil reunir la energía para hacer cosas......	0	1	2	3	4	5	6
K_5	Preocuparse en exceso es una característica de mi familia.....	0	1	2	3	4	5	6
K_6	A menudo siento que la vida no tiene mucho qué ofrecerme...	0	1	2	3	4	5	6
K_7	Aun cuando me esfuerce mucho en la preparación para una presentación, seguramente voy a cometer errores	0	1	2	3	4	5	6
K_8	Me cuesta depender de otras personas..................................	0	1	2	3	4	5	6
K_9	Mis padres han sido generalmente sensibles a mis necesidades y han respondido a ellas	6	5	4	3	2	1	0
K_10	Durante o antes de una presentación, tengo sensaciones parecidas al pánico…………………………………………………………..	0	1	2	3	4	5	6
K_11	Antes de un concierto nunca sé si mi desempeño será bueno……………………………………………………………	0	1	2	3	4	5	6
K_12	Durante o antes de una presentación, siento la boca seca…………………………………………………………	0	1	2	3	4	5	6
K_13	A menudo siento que no valgo mucho como persona.............	0	1	2	3	4	5	6
K_14	Durante un concierto me pregunto a veces si lograré llegar hasta el final de una pieza..	0	1	2	3	4	5	6
K_15	Pensar acerca de la evaluación que pueda obtener interfiere con mi desempeño	0	1	2	3	4	5	6
K_16	Durante o antes de una presentación tengo nauseas o siento que me voy a desmayar…………………………………………..	0	1	2	3	4	5	6
K_17	Aun en las presentaciones más estresantes, tengo la seguridad de que tendré un buen desempeño	6	5	4	3	2	1	0
K_18	A menudo me preocupa una reacción negativa de la audiencia	0	1	2	3	4	5	6
K_19	Algunas veces me pongo ansioso(a) sin ninguna razón aparente	0	1	2	3	4	5	6

K_20	Desde una etapa temprana de mis estudios musicales recuerdo sentir ansiedad respecto de tocar en público	0	1	2	3	4	5	6
K_21	Me preocupa que un mal concierto pueda arruinar mi carrera	0	1	2	3	4	5	6
K_22	Durante o antes de una presentación, experimento un aumento en el ritmo cardiaco como si fueran golpes en el pecho………………………………………………………………..	0	1	2	3	4	5	6
K_23	Mis padres casi siempre me escuchaban	6	5	4	3	2	1	0
K_24	Dejo pasar valiosas oportunidades de presentación	0	1	2	3	4	5	6
K_25	Después de una presentación, me preocupa si toqué lo suficientemente bien………………………………..………....	0	1	2	3	4	5	6
K_26	La preocupación y nerviosismo sobre mi desempeño interfieren con mi atención y concentración………………………………..	0	1	2	3	4	5	6
K_27	En la infancia, a menudo me sentía triste	0	1	2	3	4	5	6
K_28	A menudo me preparo para un concierto con una sensación de temor y de desastre inevitable…………………………...	0	1	2	3	4	5	6
K_29	Uno o dos de mis padres eran sumamente ansiosos……….	0	1	2	3	4	5	6
K_30	Durante o antes de una presentación, tengo mayor tensión muscular…………………………………………………………..	0	1	2	3	4	5	6
K_31	A menudo siento que no tengo muchas expectativas futuras ..	0	1	2	3	4	5	6
K_32	Después de una presentación, la repito en mi mente una y otra vez……………………………………………………………	0	1	2	3	4	5	6
K_33	Mis padres me alentaron a probar cosas nuevas	6	5	4	3	2	1	0
K_34	Me preocupo tanto por una presentación que no puedo dormir	0	1	2	3	4	5	6
K_35	Mi memoria es confiable cuando toco sin partitura……..	6	5	4	3	2	1	0
K_36	Durante o antes de una presentación me siento tembloroso(a) o tambaleante……..……………………………...	0	1	2	3	4	5	6
K_37	Tengo confianza al tocar de memoria.....................................	6	5	4	3	2	1	0
K_38	Me preocupa ser observado(a) y analizado(a) por otros……………	0	1	2	3	4	5	6
K_39	Me preocupa mi propio juicio acerca de mi desempeño……………………………………………………..….	0	1	2	3	4	5	6
K_40	Me mantengo con el compromiso de tocar aun cuando me cause gran ansiedad…………………………………………..….	0	1	2	3	4	5	6

©Kenny, D.T. (2009). *Kenny Music Performance Anxiety Inventor-Revised* (K-MPAI-R). Adaptación al español del *Kenny Music Performance Anxiety Inventor-Revised* (K-MPAI-R), Kenny, D.T. (2009), por Álvaro Mario Chang Arana, Lima, Perú, 2013.
