# Supplementary material for: The Kenny music performance anxiety inventory (K-MPAI): Scale construction, cross-cultural validation, theoretical underpinnings, and diagnostic and therapeutic utility
Source: Front Psychol. 2023 May 26;14:1143359. doi: 10.3389/fpsyg.2023.1143359 (PMC10262052; doi:10.3389/fpsyg.2023.1143359)
Supplement: Supplementary file 2 [file Data_Sheet_1.zip › K-MPAI_Taiwanese translation.pdf]

(K-MPAI-R-C)

以下是一些關於你一般感覺和**表演前或演出期間**的感覺的陳述。請圈一個數位,以表明您同意或不同意每個陳述的數量。**(強烈不同意=0,不同意=1,略有異議 = 2,中性 =3,略為同意 = 4,同意 = 5,強烈同意 = 6)**

K1	我通常覺得能夠控制我的生活	6	5	4	3	2	1	0
K2	我發現信任別人對我來說是很容易的	6	5	4	3	2	1	0
K3	有時我感到不知道為什麼而沮喪	0	1	2	3	4	5	6
K4	我經常發現很難把全副精力用在做事情上	0	1	2	3	4	5	6
K5	過度的擔心是我家裡的一個特點	0	1	2	3	4	5	6
K6	我常常覺得生活沒有太多東西可以給我充實的感覺	0	1	2	3	4	5	6
K7	即使我努力練習,我表演時很可能還是會犯錯誤	0	1	2	3	4	5	6
K8	我發現依賴別人是很困難的	0	1	2	3	4	5	6
K9	我的父母大多時候能夠回應我的需求	6	5	4	3	2	1	0
K10	在演出之前或演出期間,我有于恐慌的感覺	0	1	2	3	4	5	6
K11	在音樂會之前,我從來不知道我到時是否能表現好	0	1	2	3	4	5	6
K12	在演出之前或演出期間,我常感到口乾	0	1	2	3	4	5	6
K13	我常常覺得作為一個人並沒有特別的意義	0	1	2	3	4	5	6
K14	表演中,我發現自己常在想我是否能度過難關	0	1	2	3	4	5	6
K15	考慮別人對我的評估時可能會干擾我的表現	0	1	2	3	4	5	6
K16	在演出之前或演出期間,我感到噁心或頭昏,或胃部不舒服	0	1	2	3	4	5	6
K17	即使在壓力最大的表現情況下, 我也會有好的表現	6	5	4	3	2	1	0
K18	我經常擔心觀眾的負面反應	0	1	2	3	4	5	6
K19	有時我感到焦慮沒有特別的原因	0	1	2	3	4	5	6
K20	從我學習音樂的早期,我記得對表演感到焦慮	0	1	2	3	4	5	6

K21	我擔心一個壞的表現可能會毀了我的將來	0	1	2	3	4	5	6
K22	在演出之前或演出期間,我感覺到心率增加,胸口的跳動	0	1	2	3	4	5	6
K23	我的父母幾乎總是聽我的	6	5	4	3	2	1	0
K24	我曾放棄了有價值的表演機會	0	1	2	3	4	5	6
K25	演出結束後,我仍擔心是否自己表現是足夠好的	0	1	2	3	4	5	6
K26	我對表現的擔心和緊張干擾了我的注意力和注意力	0	1	2	3	4	5	6
K27	作為一個孩子,我經常感到悲傷	0	1	2	3	4	5	6
K28	我經常帶著恐懼和即將來臨的災難的感覺來準備音樂會	0	1	2	3	4	5	6
K29	我的父母 (其中一個或雙親)都過於焦慮	0	1	2	3	4	5	6
K30	在表演之前或演出期間,我有肌肉緊繃的情況	0	1	2	3	4	5	6
K31	我常常覺得我沒有什麼可期待的	0	1	2	3	4	5	6
K32	演出結束後,我一遍又一遍地在腦海中重播演出的情景	0	1	2	3	4	5	6
K33	我父母(其中一個或雙親)經常鼓勵我嘗試新事物	6	5	4	3	2	1	0
K34	演出前我很擔心,會睡不著覺	0	1	2	3	4	5	6
K35	在沒有樂譜的情況下表演時,我的背譜記憶是可靠的	6	5	4	3	2	1	0
K36	在演出之前或演出期間,我感覺顫抖或震顫	0	1	2	3	4	5	6
K37	我感到有信心背譜演出	6	5	4	3	2	1	0
K38	我擔心被別人評論我的演出	0	1	2	3	4	5	6
K39	我擔心自己無法對自我評價很高	0	1	2	3	4	5	6
K40	我仍然致力於表演,即使它引起我極大的焦慮	0	1	2	3	4	5	6
