# Supplementary material for: The Kenny music performance anxiety inventory (K-MPAI): Scale construction, cross-cultural validation, theoretical underpinnings, and diagnostic and therapeutic utility
Source: Front Psychol. 2023 May 26;14:1143359. doi: 10.3389/fpsyg.2023.1143359 (PMC10262052; doi:10.3389/fpsyg.2023.1143359)
Supplement: Supplementary file 2 [file Data_Sheet_1.zip › K-MPAI_Turkish translation.pdf]

Kenny Müzik Performansı Kaygı Ölçeği ve Değerlendirme Formu

Aşağıda, **bir performans esnasında veya öncesinde** genel olarak nasıl hissettiğiniz ve duygusal durumunuz hakkında bazı ifadeler yer almaktadır. Her bir ifadeye ne derece katıldığınız veya katılmadığınızı belirtmek üzere ilgili rakamı yuvarlak içine alarak işaretleyiniz.

		**Hiç Katılmıyorum**						**Tamamen Katılıyorum**
K_1	Genelde, hayatımın kontrolüm altında olduğunu hissediyorum........	6	5	4	3	2	1	0
K_2	Başkalarına rahatlıkla güveniyorum........	6	5	4	3	2	1	0
K_3	Bazen sebebini bilmediğim halde kendimi kötü hissediyorum........	0	1	2	3	4	5	6
K_4	Genelde bir şeyler yapma enerjisinden yoksun oluyorum..	0	1	2	3	4	5	6
K_5	Aşırı endişe, ailemin tipik özelliğidir........	0	1	2	3	4	5	6
K_6	Çoğu zaman hayatın bana verecek fazla bir şeyi olmadığını hissediyorum........	0	1	2	3	4	5	6
K_7	Bir performansa hazırlanmak için çok çalışsam da, hata yapma olasılığım yüksek oluyor........	0	1	2	3	4	5	6
K_8	Başkalarına güvenmekte zorlanıyorum........	0	1	2	3	4	5	6
K_9	Ailem, ihtiyaçlarım karşısında genelde duyarlıdır........	6	5	4	3	2	1	0
K_10	Bir performans sırasında veya öncesinde, panik benzeri duygular yaşıyorum........	0	1	2	3	4	5	6
K_11	Bir konserden önce, iyi bir performans çıkarıp çıkaramayacağımdan hiçbir zaman emin olamıyorum........	0	1	2	3	4	5	6
K_12	Bir performans sırasında veya öncesinde ağzım kurur.......	0	1	2	3	4	5	6
K_13	Genelde, bir insan olarak pek de değerli olmadığımı hissediyorum........	0	1	2	3	4	5	6
K_14	Bir performans sırasında, bu işin altından kalkıp kalkamayacağımı düşündüğümü fark ediyorum........	0	1	2	3	4	5	6
K_15	Değerlendirme kısmını düşündüğümde, performansımda sorunlar yaşayabiliyorum........	0	1	2	3	4	5	6
K_16	Bir performans sırasında veya öncesinde, kendimi hasta veya fenalaşacak veya midem kasılıyormuş gibi hissediyorum........	0	1	2	3	4	5	6
K_17	En stresli performanslar sırasında bile, iyi bir performans çıkaracağım konusunda kendime güveniyorum........	6	5	4	3	2	1	0
K_18	Genelde, seyirciden olumsuz bir tepki gelmesinden endişe ederim........	0	1	2	3	4	5	6
K_19	Bazen, herhangi bir sebep olmaksızın endişeli hissederim.	0	1	2	3	4	5	6
K_20	Müzik çalışmalarımın erken dönemlerinden itibaren performans konusunda endişe duyduğumu hatırlıyorum..	0	1	2	3	4	5	6

İşbu tercüme İngilizce aslından Türkçeye tarafımdan yapılmıştır.
Ordu 3. Noteri Yeminli Tercümanı
Funda ALTIN

K_21	Kötü bir performansın, kariyerimi mahvedeceğinden korkuyorum......	0	1	2	3	4	5	6
K_22	Bir performans sırasında veya öncesinde kalbim sanki göğsümden fırlayacakmış gibi hızlı atıyor......	0	1	2	3	4	5	6
K_23	Ailem neredeyse her zaman söylediklerimi dinlemiştir......	6	5	4	3	2	1	0
K_24	Kayda değer performans olanaklarından umudumu kestim......	0	1	2	3	4	5	6
K_25	Performans sonrasında, yeterince iyi çalıp çalmadığım konusunda endişe duyarım......	0	1	2	3	4	5	6
K_26	Performansım hakkında hissettiğim endişe ve tedirginlik, odaklanmama ve konsantre olmama engel oluyor......	0	1	2	3	4	5	6
K_27	Çocukken, çoğunlukla üzgün hissederdim......	0	1	2	3	4	5	6
K_28	Bir konsere hazırlanırken içimde genelde korku ve yaklaşan felaket hissi olur......	0	1	2	3	4	5	6
K_29	Ebeveynlerimden biri veya her ikisi son derece endişeli bir mizaca sahipti......	0	1	2	3	4	5	6
K_30	Bir performans sırasında veya öncesinde, gittikçe şiddetlenen kas kasılmaları yaşarım......	0	1	2	3	4	5	6
K_31	Genellikle herhangi bir şeye karşı yoğun isteklerim olmadığını hissediyorum......	0	1	2	3	4	5	6
K_32	Bir performansın ardından, onu kafamın içinde tekrar tekrar çalıyorum......	0	1	2	3	4	5	6
K_33	Ailem beni yeni şeyler denemek konusunda teşvik eder....	6	5	4	3	2	1	0
K_34	Bir performans öncesinde o kadar çok kaygılanırım ki uyuyamam......	0	1	2	3	4	5	6
K_35	Müziksiz performans gerçekleştirdiğimde hafızama güvenirim......	6	5	4	3	2	1	0
K_36	Bir performans sırasında veya öncesinde, sarsıntı veya titreme veya çarpıntı hissederim......	0	1	2	3	4	5	6
K_37	Bir eseri hafızadan çalma konusunda kendime güvenirim..	6	5	4	3	2	1	0
K_38	Başkalarının dikkatlerinin üzerimde olmasından endişe duyarım......	0	1	2	3	4	5	6
K_39	Bir performansımı nasıl gerçekleştireceğimi düşündüğümde, kendi yargılarımdan endişe duyarım......	0	1	2	3	4	5	6
K_40	Beni çok fazla endişelendirse de performans gerçekleştirme olgusuna bağlıyımdır......	0	1	2	3	4	5	6

İşbu tercüme İngilizce aslından Türkçeye tarafımdan yapılmıştır.
Ordu 3. Noteri Yeminli Tercümanı
Funda ALTIN
