# Supplementary material for: The Kenny music performance anxiety inventory (K-MPAI): Scale construction, cross-cultural validation, theoretical underpinnings, and diagnostic and therapeutic utility
Source: Front Psychol. 2023 May 26;14:1143359. doi: 10.3389/fpsyg.2023.1143359 (PMC10262052; doi:10.3389/fpsyg.2023.1143359)
Supplement: Supplementary file 2 [file Data_Sheet_1.zip › K-MPAI_Ukrainian translation.pdf]

Опитувальник сценічної тривоги музикантів Д. Кенні
Kenny Music Performance Anxiety Inventory (K-MPAI)

Нижче наведено твердження про те, як ви почуваєтеся загалом і як ви почуваєтеся перед або під час виступу. Обведіть, будь ласка, число, яке вказує, наскільки ви згодні чи не згодні з кожним твердженням.

		**Зовсім не згідний/-на**						**Повністю згідний/-на**
1	Я загалом відчуваю контроль над власним життям	6	5	4	3	2	1	0
2	Я легко довіряю іншим	6	5	4	3	2	1	0
3	Часом почуваю себе пригнічено без вагомих причин	0	1	2	3	4	5	6
4	Мені часто важко відчути достатній рівень енергії, щоб щось зробити	0	1	2	3	4	5	6
5	Надмірна тривожність – це риса властива моїй сім'ї	0	1	2	3	4	5	6
6	Я часто відчуваю, що не надто багато отримую від життя	0	1	2	3	4	5	6
7	Навіть коли докладаю багато зусиль у підготовці до виступу, я все ж схильний/-а помилятися	0	1	2	3	4	5	6
8	Мені важко залежати від інших	0	1	2	3	4	5	6
9	Батьки в більшості випадків турбувалися про задоволення моїх потреб	6	5	4	3	2	1	0
10	Перед або під час виступу в мене виникають переживання, схожі на паніку	0	1	2	3	4	5	6
11	Перед концертом я ніколи не знаю чи виступлю добре	0	1	2	3	4	5	6
12	До або під час виступу я відчуваю сухість у роті	0	1	2	3	4	5	6
13	Я часто відчуваю, що нічого не вартий/-а як людина	0	1	2	3	4	5	6
14	Під час виступу я ловлю себе на думці про те, чи зможу це зробити	0	1	2	3	4	5	6
15	Думка про те, що інші мене оцінюють, заважає моєму виконанню	0	1	2	3	4	5	6
16	Перед або під час виступу я почуваю нудоту, слабкість або спазми у животі	0	1	2	3	4	5	6
17	Навіть у найбільш стресових ситуаціях на сцені, я впевнений/-а, що виступлю добре	6	5	4	3	2	1	0
18	Мене часто турбує негативна реакція слухачів	0	1	2	3	4	5	6

		Зовсім не згідний/-на						Повністю згідний/-на
19	Часом почуваюся тривожно без особливих на те причин	0	1	2	3	4	5	6
20	Я пам’ятаю, що від початку навчання музиці завжди тривожився/-лася через виступи	0	1	2	3	4	5	6
21	Я переживаю, що один поганий виступ може зруйнувати мою кар’єру	0	1	2	3	4	5	6
22	До або під час виступу я відчуваю пришвидшене серцебиття та стукіт у грудях	0	1	2	3	4	5	6
23	Батьки майже завжди прислухалися до моєї думки	6	5	4	3	2	1	0
24	Я відмовляюся від важливих можливостей виступати	0	1	2	3	4	5	6
25	Після виступу я непокоюся чи все виконав/-ла достатньо добре	0	1	2	3	4	5	6
26	Пов’язані з виступом неспокій та нервозність відволікають та заважають зосередитися	0	1	2	3	4	5	6
27	В дитинстві я часто почувався/ лася сумним/-ою	0	1	2	3	4	5	6
28	Я часто готуюся до концерту з відчуттям страху та неминучої катастрофи	0	1	2	3	4	5	6
29	Один або обоє моїх батьків були надмірно тривожними	0	1	2	3	4	5	6
30	До або під час виступу в мене зростає напруга у м’язах	0	1	2	3	4	5	6
31	Я часто відчуваю, що мені немає на що сподіватися у майбутньому	0	1	2	3	4	5	6
32	Після виступу я знову і знову прокручую його у голові	0	1	2	3	4	5	6
33	Мої батьки заохочували мене пробувати щось нове	6	5	4	3	2	1	0
34	Я так сильно переживаю перед виступом, що не можу спати	0	1	2	3	4	5	6
35	У справах, не пов’язаних з музикою, моя пам'ять є надійною	6	5	4	3	2	1	0
36	До або під час виступу я відчуваю тремтіння або тремор	0	1	2	3	4	5	6
37	Я почуваюся впевнено, коли граю напам’ять	6	5	4	3	2	1	0
38	Мені ніяково, коли інші за мною уважно спостерігають	0	1	2	3	4	5	6
39	Мене непокоїть моя особиста оцінка мого виступу	0	1	2	3	4	5	6
40	Я продовжую виступати, не зважаючи на сильну тривогу	0	1	2	3	4	5	6
