# Supplementary material for: The Kenny music performance anxiety inventory (K-MPAI): Scale construction, cross-cultural validation, theoretical underpinnings, and diagnostic and therapeutic utility
Source: Front Psychol. 2023 May 26;14:1143359. doi: 10.3389/fpsyg.2023.1143359 (PMC10262052; doi:10.3389/fpsyg.2023.1143359)
Supplement: Supplementary file 2 [file Data_Sheet_1.zip › KMPAI_Japanese translation.pdf]

以下は、一般的な心境と、演奏前・演奏中の心境についての文章です。それぞれの文章について１つの数字を選び、どの程度あなたにあてはまるかを答えてください。

		全く あてはまらない						非常に あてはまる
K_1	概して自分の人生をコントロールしていると感じる。...................	6	5	4	3	2	1	0
K_2	他人を信頼することは簡単だと思う。.................................	6	5	4	3	2	1	0
K_3	訳も分からず落ち込むことが時々ある。................................	0	1	2	3	4	5	6
K_4	物事を行う元気を出しづらいと思うことがよくある。....................	0	1	2	3	4	5	6
K_5	自分の家族は心配し過ぎる傾向がある。...................................	0	1	2	3	4	5	6
K_6	人生から得るものはあまりないと感じることがよくある。.....................	0	1	2	3	4	5	6
K_7	演奏に向けて懸命に準備しても、ミスをしがちである。....................	0	1	2	3	4	5	6
K_8	他人に頼ることが苦手だと思う。..	0	1	2	3	4	5	6
K_9	自分の両親は、ほぼ自分の要求に応えてくれた。..........................	6	5	4	3	2	1	0
K_10	演奏前や演奏中に、パニックに近い感覚になる。..........................	0	1	2	3	4	5	6
K_11	コンサート前は、自分がうまく演奏できるかどうか全く分からない。......	0	1	2	3	4	5	6
K_12	演奏前や演奏中に、口の渇きを経験する。...........................	0	1	2	3	4	5	6
K_13	自分はあまり価値のない人間だと感じることがよくある。................	0	1	2	3	4	5	6
K_14	演奏中に、自分がやり通せるかどうかとさえ考えてしまう。................	0	1	2	3	4	5	6
K_15	自分が受けるかもしれない評価のことを考えると、自分の演奏が妨げられる。..	0	1	2	3	4	5	6
K_16	演奏前や演奏中に、気分が悪くなったり、気が遠くなったり、胃がむかむかしたりする。..	0	1	2	3	4	5	6
K_17	最もストレスがかかる演奏場面でさえ、自分はうまく演奏できる自信がある。...	6	5	4	3	2	1	0
K_18	聴衆のネガティブな反応が気になることがよくある。.........................	0	1	2	3	4	5	6
K_19	特に訳もなく不安を感じることが時々ある。..................................	0	1	2	3	4	5	6
K_20	音楽を学び始めたばかりの頃から、演奏することに不安を感じていたのを覚えている。..	0	1	2	3	4	5	6

		全く<br>あてはまらない						非常に<br>あてはまる
K_21	一度の演奏の失敗で自分のキャリアが台無しになるかもしれないと心配になる。	0	1	2	3	4	5	6
K_22	演奏前や演奏中に、胸がドキドキするような心拍数の増加を経験する。	0	1	2	3	4	5	6
K_23	自分の両親はほぼいつも自分の言うことを聞いてくれた。	6	5	4	3	2	1	0
K_24	やりがいのある演奏の機会を諦める。	0	1	2	3	4	5	6
K_25	演奏後に、自分が十分うまく演奏できたかどうか心配になる。	0	1	2	3	4	5	6
K_26	自分の演奏に関する心配と緊張によって、集中が妨げられる。	0	1	2	3	4	5	6
K_27	子供の頃、悲しい思いをすることがよくあった。	0	1	2	3	4	5	6
K_28	恐怖と、災難が迫り来るような感覚でコンサートの準備をすることがよくある。	0	1	2	3	4	5	6
K_29	自分の片親または両親は、不安になり過ぎる傾向があった。	0	1	2	3	4	5	6
K_30	演奏前や演奏中に、筋肉の緊張が増す。	0	1	2	3	4	5	6
K_31	楽しみにすることが何もないと感じることがよくある。	0	1	2	3	4	5	6
K_32	演奏後、何度も繰り返し演奏を思い起こす。	0	1	2	3	4	5	6
K_33	自分の両親は新しい物事に挑戦するよう勧めてくれた。	6	5	4	3	2	1	0
K_34	演奏前は、心配し過ぎて眠れない。	0	1	2	3	4	5	6
K_35	暗譜で演奏する際、自分の記憶力は頼りになる。	6	5	4	3	2	1	0
K_36	演奏前や演奏中に、身体の震えを経験する。	0	1	2	3	4	5	6
K_37	暗譜で演奏することに自信がある。	6	5	4	3	2	1	0
K_38	他人にじっと見られると気になる。	0	1	2	3	4	5	6
K_39	自分がどの程度うまく演奏できると思えるかが気になる。	0	1	2	3	4	5	6
K_40	演奏することで強い不安を感じるが、演奏に全力を注いでいく。	0	1	2	3	4	5	6

©Kenny, D.T. (2009). *Kenny Music Performance Anxiety Inventory-Revised* (K-MPAI-R)
Translated by Sakie Takagi, Michiko Yoshie, and Akihiko Murai
